# Brain network reconfiguration during reward prediction error processing

**DOI:** 10.1162/NETN.a.544

**Published:** 2026-08-28

**Authors:** Kamil Bonna, Oliver J. Hulme, Simon R. Steinkamp, David Meder, Maria E. C. van der Weij, Włodzisław Duch, Karolina Finc

**Affiliations:** Centre for Modern Interdisciplinary Technologies, Nicolaus Copernicus University in Toruń, Toruń, Poland; Institute of Cognitive Science, Faculty of Philosophy and Social Sciences, Nicolaus Copernicus University in Toruń, Toruń, Poland; Danish Research Centre for Magnetic Resonance, Department of Radiology and Nuclear Medicine, Copenhagen University Hospital - Amager and Hvidovre, Copenhagen, Denmark; London Mathematical Laboratory, London, UK; Department of Psychology, University of Copenhagen, Copenhagen, Denmark; Department of Informatics, Institute of Engineering and Technology, Faculty of Physics, Astronomy and Informatics, Nicolaus Copernicus University in Toruń, Toruń, Poland; Institute of Advanced Studies, Centre for Modern Interdisciplinary Technologies, Nicolaus Copernicus University in Toruń, Toruń, Poland

**Keywords:** Decision making, fMRI, Network reconfiguration, Modularity, Reward prediction error, RPE, Reinforcement learning

## Abstract

Learning from experience is theorized to be driven by reward prediction error (RPE) signals that reflect updates to our expectations of reward. Despite numerous studies on the neural correlates of RPEs, the question of how large-scale networks (LSN) in the brain reconfigure in response to an RPE learning signal remains open. Here, we examine how functional networks change in response to RPEs depending on the context. In our study, participants performed a probabilistic reversal learning task while we acquired fMRI data in two experimental settings: reward-seeking and punishment-avoiding. Participants’ behavior was best explained by models with different learning rates for positive and negative RPEs. Furthermore, no evidence was found for context-dependent learning rates. Using behaviorally fitted RPE models, we performed a whole-brain network analysis. This analysis revealed classical reward structures, where striatal reward networks emerge as modules when the community structure is examined at a finer resolution, and a ventromedial prefrontal network emerges at a coarser resolution. Using the same behavioral model, we found that, compared with negative RPEs, positive RPEs increased within-network integration and decreased between-community integration. This indicates that there are distinctly different neural processes for positive and negative RPEs.

## Author Summary

Learning from experience is driven by reward prediction error (RPE) signals—the difference between what we expect and what we actually experience. While the neural regions involved in RPE processing are well established, it remains unclear how large-scale brain networks reorganize to support learning. We addressed this question by combining computational modeling with fMRI while participants performed a probabilistic learning task in two contexts: seeking rewards and avoiding punishments. We found that behavioral learning rates depended on whether an outcome was better or worse than expected, but not on whether it involved reward or punishment. Consistent with this behavioral pattern, network analyses showed that positive RPEs were associated with increased segregation of brain networks, including the emergence of a distinct ventromedial prefrontal cortex module. In contrast, negative RPEs led to increased integration between default mode, sensory, and visual networks. Together, these findings suggest that brain network reconfiguration is primarily organized by the sign of the prediction error rather than by outcome valence. Negative prediction errors appear to recruit a more globally integrated and cognitively demanding network state, whereas positive errors rely on more segregated and specialized subsystems.

## INTRODUCTION

Learning through interaction with the environment is an essential feature of all intelligent systems. Reinforcement learning (RL) is a computational framework in which agents learn to make decisions by maximizing the expected cumulative rewards over time (Sutton & Barto, 2018). Rewarding events typically reinforce behavior, whereas punishing events have the opposite effect. Learning occurs by comparing the predicted reward following an action or stimulus with the actually experienced reward. The difference between the two, the reward prediction error (RPE), plays a critical role in updating reward expectation (sometimes called the value).

Remarkably, empirical findings suggest that the brain encodes RPEs by modulating the phasic activity of dopaminergic neurons in the midbrain (Colombo, 2014; Montague et al., 1996), which project throughout the entire brain, engaging interconnected networks. Despite extensive investigations into the neural correlates of RPEs, which are often restricted to specific brain regions, the impact of such coding on the entire brain network remains an important but unaddressed question. In this study, we investigate—on a network level—how implementations of the RPE signaling in reward-seeking and punishment-avoiding contexts differ and how these signals affect network configurations.

RPE signals are bivalent, allowing learning from both punishing and rewarding events. Independent of the outcome, a positive RPE encodes a better-than-expected outcome (more reward, less punishment), and a negative RPE encodes a worse-than-expected outcome (less reward, more punishment). Most studies investigating reward learning here focus on the signed RPE as a whole, which, according to a meta-analysis, predominantly correlates with brain activity in the striatum, putamen, anterior cingulate cortex, and ventromedial prefrontal cortex (vmPFC; Fouragnan et al., 2018). The same meta-analysis, however, also investigated the sign (the valence) of the RPE, identifying two spatially distinct learning networks for processing positive and negative RPEs (Fouragnan et al., 2018). The network coding positive RPEs involved regions such as ventrolateral prefrontal cortex, ventral striatum (VS), and posterior cingulate cortex, while the network coding negative RPEs involved regions such as dorsomedial cingulate cortex, anterior insula, pallidum (PA), and middle frontal gyrus. A similar observation can be made when investigating punishing and rewarding contexts. While the computational theory behind RPEs does not distinguish between contexts, the brain does. There seems to be clear convergence about the encoding of rewards (VS, etc.), whereas punishments are encoded more broadly, showing that both classic dopaminergic brain networks are activated, as well as regions outside of it (Palminteri & Pessiglione, 2017).

Knowing that the brain reacts differently to punishment and reward, we also want to understand how this affects learning in these contexts. For example, for some time, it has been discussed how the reward system could effectively implement learning in punishing contexts as successful avoidance, since these events would cease to trigger dopamine release after a few occurrences, based on the RPE theory. However, the reference point theory seems to alleviate this issue. By taking the average reward of an event or context into account, successful avoidance can still trigger a reinforcing signal (a positive RPE). For example, when all outcomes are losses, an outcome that is less bad than the average would evoke a positive RPE. Such context dependence may suggest that the brain uses a *reference point* to which experienced outcomes are compared (Bavard et al., 2018; Rangel & Clithero, 2012). The reference hypothesis was supported by both behavioral (Khaw et al., 2017; Louie et al., 2013) and neuroimaging studies using functional magnetic resonance imaging (fMRI; Rigoli, 2019). Investigating the interplay between the sign of the RPE and the context, studies have found that the ventral reward system signaled positive RPEs irrespective of the outcome valence (Meder et al., 2016; Palminteri et al., 2015). On the other hand, the amygdala, inferior frontal gyrus, and dorsomedial prefrontal cortex signaled positive RPEs in a reward context but not in a punishment context (Meder et al., 2016). Due to these inconclusive findings, context dependence of RPE coding is still a debated issue. Moreover, it is unknown whether the reference effect is also reflected by the outcome valence invariance at the whole-brain network level.

In recent years, the interdisciplinary field of *network neuroscience* (Bassett & Sporns, 2017) has provided valuable insights into the dynamic nature of functional brain networks, highlighting their capacity for reorganization in response to environmental demands (Bullmore & Sporns, 2009). We quantify LSN organization using agreement, which captures the probability that regions belong to the same network community. High within- and low between-LSN agreement indicates stronger segregation, reflecting distinct and stable network communities, whereas higher between-LSN agreement reflects greater integration across networks. To date, there have been few attempts to investigate LSNs in the brain from the perspective of RL. Most studies investigated gradual network changes associated with reward learning and RPEs, but did not focus on the RPE’s sign (Gerraty et al., 2018; Mattar et al., 2018). Others, such as Sadler et al. (2020), used a taste-motivated learning task to examine the dynamics of the reward network and found that the community structure of the functional brain networks was affected by the outcome—ventromedial and ventrolateral prefrontal cortices changed their module assignment with a sweet taste compared with a bitter taste. This limited evidence leaves the relationship between whole-brain network restructuring dynamics and RPE encoding largely unexplored.

A few studies have investigated context dependence (reward vs. punishment) of connectivity profiles during RPE processing. In one study, Camara et al. (2009) investigated functional connectivity patterns during monetary gain and losses. For example, a network of brain regions encompassing the amygdala, orbitofrontal cortex, and insula was coupled with the VS during monetary gambling irrespective of the outcome valence. However, the orbitofrontal cortex had stronger connections with the VS during punishing trials. Other studies found increased functional connectivity between the VS and vmPFC as well as between the amygdala and VS, midbrain, cingulate cortex, thalamus, orbitofrontal cortex, and dorsolateral prefrontal cortex during reward processing (Cohen et al., 2005; van den Bos et al., 2012). Overall, these findings yield inconsistent answers as to the question of whether regions encoding RPEs share similar or different connectivity profiles during reinforcement reward and punishment processing.

The main goal of this work is to provide a comprehensive description of the LSN reconfiguration during RPE processing under both reward and punishment contexts. We tasked 32 healthy participants with playing a probabilistic reversal learning (PRL) paradigm, in both reward-seeking and punishment-avoiding contexts, while acquiring whole-brain fMRI data. First, we modeled participants’ behavior with a Bayesian cognitive model with four competing submodels and assessed which computational model of RL best explains the subjects’ decisions. Then, we used the parameters of the best fitting model to calculate the RPEs, which were used to investigate how the organization of the brain network shifts when participants switch between the processing of positive and negative RPEs. As the functional brain network is known to exhibit a multiscale community structure (Betzel & Bassett, 2017), we have focused on describing modular network reorganization on multiple topological scales—expressed by the scale parameter of the community detection algorithm. This allowed us to capture effects that might have been overlooked in a single-scale analysis (Betzel et al., 2017). Here, we had three hypotheses: (a) Switching between positive and negative RPEs will lead to dynamic reorganization of the brain network and the formation of prediction-error-specific subnetworks; (b) regions signaling RPEs will form distinct subnetworks detectable at finer topological scales; and (c) RPE-context-specific subnetworks will interact with other LSNs involved in cognitive task processing, especially the default mode network and the frontoparietal network regions; the impact of such coding on the entire brain network remains an important but unaddressed question. In this study, we investigate—on a network level—how implementations of the RPE signaling in reward-seeking and punishment-avoiding contexts differ and how these signals affect network configurations.

## RESULTS

### Behavioral Modeling of RPE Coding in Reward-Seeking and Punishment-Avoiding Contexts

**Figure 1.**
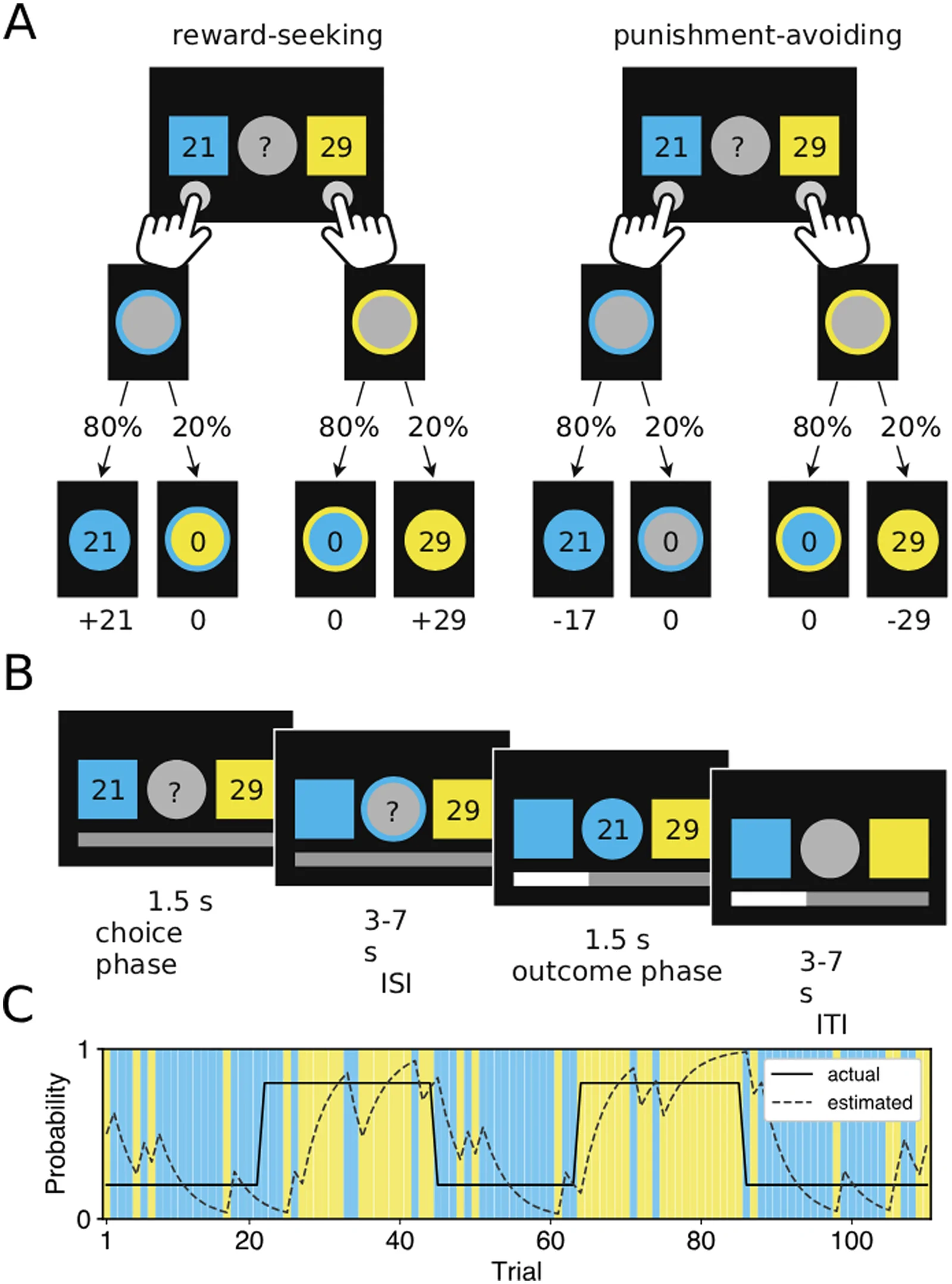
The PRL task. (A) The task structure for reward-seeking and punishment-avoiding contexts of the PRL task, represented as a decision tree. In the punishment-avoiding context, numbers in the boxes represented punishment magnitudes, the fixation circle changed color according to the chosen box, and the number of lost points was displayed in the middle. (B) Example trial of the PRL task in the reward-seeking context. Each trial began with a choice phase where the subject had 1.5 s to choose between blue and yellow boxes. Subjects had to consider both reward magnitudes visible on the boxes and reward probabilities estimated from previous trials. After a variable ISI, an outcome phase began, giving the subjects their choice feedback. During the outcome phase, the fixation circle changed color according to the chosen box, and the number of rewarded points was displayed in the middle. The outcome phase was followed by a variable ITI, after which a new trial began. (C) Each task context consisted of 110 trials. Reward contingency changed four times throughout the task. The solid line represents the true reward probability for the yellow box, whereas the dashed line represents the prediction of a standard temporal difference model.

To study RPE coding in different contexts, we used a PRL task (Figure 1; Cools et al., 2002). The PRL task is a paradigm for investigating the process of updating and relearning the stimulus–reward association through reinforcement. Here, participants performed the PRL task in two contexts: reward-seeking and punishment-avoiding. In each context, winning and losing are opposed to neutral outcomes, enabling disentangling between often confused dimensions: outcome valence and RPE sign (Palminteri & Pessiglione, 2017). Outcome valence refers to the intrinsic value of an outcome as positive or negative, whereas RPE sign indicates whether the outcome is better or worse than expected under the learning process. This quality is crucial to investigate how these dimensions independently influence decision behavior and neural correlates of RPEs.

An individual subject’s performance accuracy was calculated to establish if subjects succeeded in learning probabilistic associations. Accuracy was defined as the proportion of choices that led to rewarding outcomes in the reward-seeking context or nonpunishing outcomes in the punishment-avoiding context. Since the study examined healthy subjects without valence-dependent learning deficits, we hypothesized similar performance levels for both task contexts.

The accuracy was significantly higher than chance level for both the reward-seeking (acc_RS_ = 62.81%; one-sided *t* test, *t*(31) = 13.00; *p* < 0.0001) and the punishment-avoiding context (accPA = 62.13%; one-sided *t* test, *t*(31) = 11.38; *p* < 0.0001). Subjects performed equally well in both experimental contexts—no significant difference in performance between task contexts was found (paired *t* test; *t*(31) = 0.50, *p* = 0.62). Moreover, the number of choice reversals was calculated for each subject and task context. Subjects frequently reversed their choices—the mean number of reversals was equal to 30.41 ± 11.93 in the reward-seeking and 30.25 ± 11.98 in the punishment-avoiding context. Moreover, the number of choice reversals did not significantly differ between the two task contexts (paired *t* test; *t*(31) = 0.07, *p* = 0.94).

Finally, we quantified differences in reaction time (RT) between reward-seeking and punishment-avoiding contexts, as well as between positive and negative RPEs. RTs following rewarding/nonpunishing trials were assigned to the +RPE condition, whereas nonrewarding/punishing trials were assigned to the −RPE condition. A two-way repeated-measures ANOVA (rmANOVA) was performed on RT values with the task context and the RPE sign as factors. There was a significant interaction effect between task context and RPE sign, as measured by the *F* statistics, *F*(31) = 14.06; *p* < 0.001 (Figure 1A–B) indicating that RTs were modulated by both experimental factors. In general, RTs were longer for the punishment-avoiding compared with reward-seeking contexts, as indicated by the significant task-context effect (RT_RS_ = 687.4 ± 126.3 ms; RT_PA_ = 651.7 ± 110.9 ms; *F*(31) = 4.27; *p* < 0.05). Post hoc within-context analysis showed that RTs following negative RPEs were longer in the punishment-avoiding context (paired *t* test; *t*(31) = 3.04; *p* < 0.01) but not in the reward-seeking context (paired *t* test; *p* = 0.26).

Next, we built a Bayesian hierarchical latent-mixture model (HLM) to perform model selection and parameter estimation (see Supporting Information Section 1.1). For parameter estimation of behaviorally relevant parameters, this type of model contains learning rates and precision. For model comparisons, the model also contains indicator parameters that reflect the (sub)model probabilities. Following previous studies, models with separate learning rates for positive and negative RPEs were considered to account for possible risk-sensitivity effects. These models are referred to as prediction-error-dependent models (Niv et al., 2012; Reiter et al., 2016; van den Bos et al., 2012). To capture possible outcome valence effects, models with separate learning rates for reward-seeking and punishment-avoiding contexts were also included. These models are referred to as context-dependent models. To fully explore our model space, the HLM was set up to include all possible combinations of models. This resulted in four different models: (a) a prediction independent context independent (PICI) model with a single learning rate independent of the sign of the RPE and outcome valence, (b) a prediction independent context dependent (PICD) model with two separate learning rates for reward-seeking and punishment-avoiding contexts, (c) a prediction dependent context independent (PDCI) model with two separate learning rates for positive and negative RPEs, (d) a prediction dependent context dependent (PDCD) model with four separate learning rates for the RPE signs and task contexts. The HLM can thus be considered a supermodel that includes an indicator variable for selecting which of these four submodels explains the data best.

The HLM revealed that a majority of the subjects (27/32; 84.4%) had most of the probability mass located over either the PDCI model (19/32; 59.4%) or PDCD model (8/32, 25%; Supporting Information Figure S2A). Models with separate learning rates for reward-seeking and punishment-avoiding contexts were favored in five subjects (1/32, 3.1%; PICD and 4/32; 12.5% PICI). Posterior distribution of the model indicator variable was marginalized over subjects and model classes to investigate whether subject response patterns are better explained by models with: (a) RPE-dependent versus RPE-independent learning rates and (b) context-dependent versus context-independent learning rates. These comparisons reflected two orthogonal experimental axes: RPE sign and task context. Moderate evidence was found in favor of the hypothesis that behavioral responses are better explained by the models with separate learning rates for positive and negative RPEs (Bayes factor comparing the context-dependent vs. context-independent models [BF_PD−PI_] = 3.19; Supporting Information Figure S2B). On the other hand, there was weak evidence against the hypothesis assuming separate learning rates for reward-seeking and punishment-avoiding contexts (Bayes Factor prediction-dependent vs. prediction-independent [BF_CD−CI_] = 0.67; Supporting Information Figure S2C). Protected exceedance probabilities were computed, quantifying the probability that each submodel is the most frequent in the population, based on the marginal likelihoods across subjects. This procedure determined a single winning submodel for further fMRI analysis. The PDCI model was the most likely model across participants with a probability close to 1 (protected exceedance probability; *p* = 0.9995; Supporting Information Figure S2D).

### Functional Networks Involved in RPE Processing

Recent research identified two brain systems responsible for coding positive and negative RPEs (Fouragnan et al., 2018). How do these two brain systems interact between themselves and with other subsystems during RL in reward-seeking and punishment-avoiding contexts?

First, we applied a standard general linear model (GLM) analysis (see Supporting Information Section 2.2) to investigate the activation patterns during the processing of positive and negative RPEs in different contexts. We identified two sets of regions associated with positive and negative RPE processing, which were consistent with previous findings from the literature (Meder et al., 2016; see Supporting Information Figure S4 and Supporting Information Table S1). Furthermore, the bilateral visual areas V3 and V4, right supramarginal gyrus, right superior parietal lobule, right precuneus, right primary visual cortex, and right precentral gyrus showed higher activity during reward-seeking compared with punishment-avoiding context.

**Figure 2.**
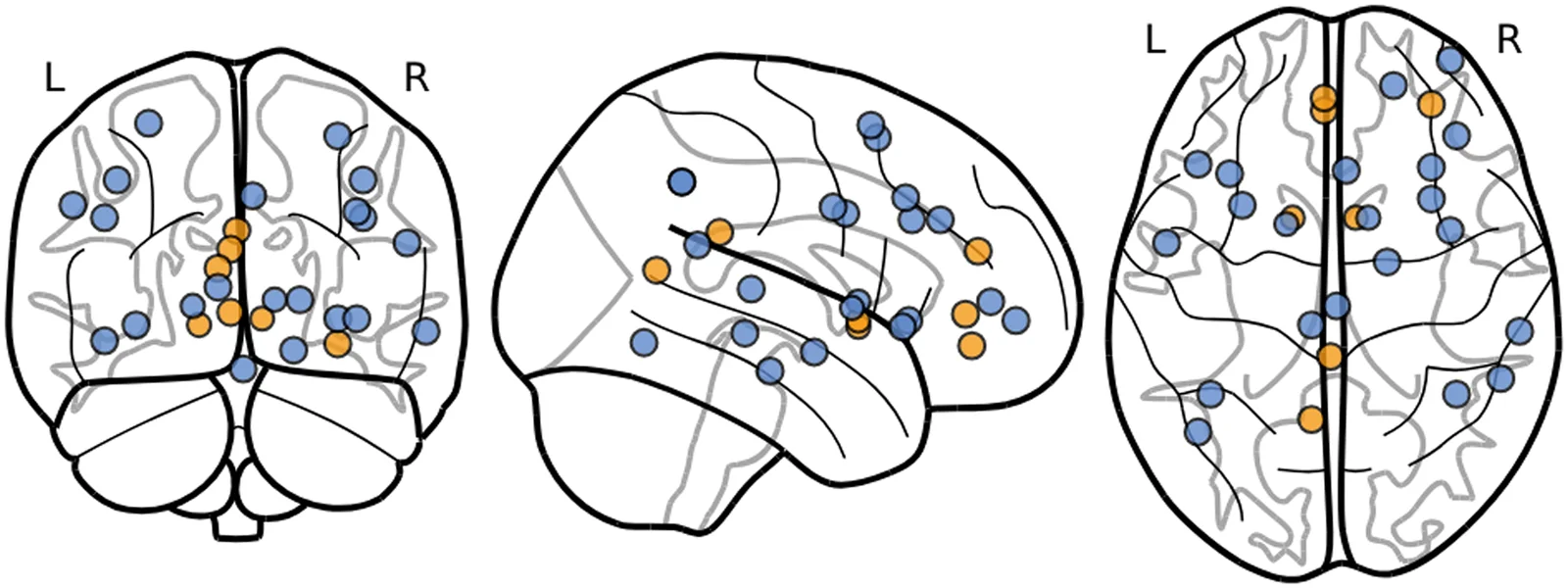
The ROIs for RPE signaling. ROIs for RPE signaling were defined based on activation likelihood estimation (ALE) meta-analysis clusters reported by Fouragnan et al. (2018). The network associated with positive RPE signaling (orange spheres) includes regions showing greater BOLD activation for positive compared with negative RPEs. Conversely, the negative RPE network (blue spheres) comprises regions with stronger BOLD responses to negative than to positive RPEs.

Then, to investigate how brain regions encoding positive and negative RPEs interact with other subsystems at the network level, we used a beta-series correlation analysis. Here, the beta weights are estimated per trial and then later sorted into positive and negative RPEs. First, we extended a standard parcellation introduced by Power et al. (2011), examining 264 regions of interest (ROIs), to include brain regions identified in a recent meta-analysis on neural representations of RPE valence (Fouragnan et al., 2018; Figure 2, Supporting Information Section 2.3.1). This procedure allowed us to study effects specific for networks signaling RPEs while still preserving reference to the well-known LSNs in the brain. Afterward, we used parameters of the best fitting behavioral model (PDCI) to investigate how the organization of the brain network shifts when participants switch between the processing of positive and negative RPEs, testing the hypothesis that there are two separate networks responsible for positive and negative RPE processing.

Instead of only testing for context-related regional activity changes, exploring the pattern of changes in network segregation can provide additional insights into how the brain achieves learning and decision-making in different contexts. Here, we used an approach based on within- and between-community agreement to investigate whether the integration and segregation of LSNs depend on the RPE sign and outcome valence. This community-level approach relies on averaging the node-level agreement (*D*_*ij*_) derived from data-driven partitions using a reference partition. The reference partition can be either a consensus partition or an a priori partition representing a stable community structure (Conrad et al., 2020). To better reflect the underlying structure of the data, the context-independent consensus partitions were used as reference partitions (see Figure 7). Community-level agreement values capture the level of association between LSNs; when calculated for a single partition, they represent a subject-level pattern of integration and segregation of different brain systems.

*Within-community agreement* expresses the probability that two randomly selected nodes from a given LSN (defined by the consensus partition) will be found within the same (subject-level) data-driven community. For example, a within-community agreement of one would reflect that all LSN nodes are part of the same data-driven community. High values of within-community agreement indicate stable and integrated LSNs, whereas lower values characterize more disorganized and unstable systems. *Between-community agreement* reflects the probability that a randomly selected pair of nodes from two different LSNs will share the same data-driven community. In most extreme cases, the between-community agreement can be zero when all nodes from both LSNs belong to different data-driven communities, or it could be one when both LSNs are merged into a larger community in a data-driven partition. Between-community agreement expresses the extent of integration between LSNs.

**Figure 3.**
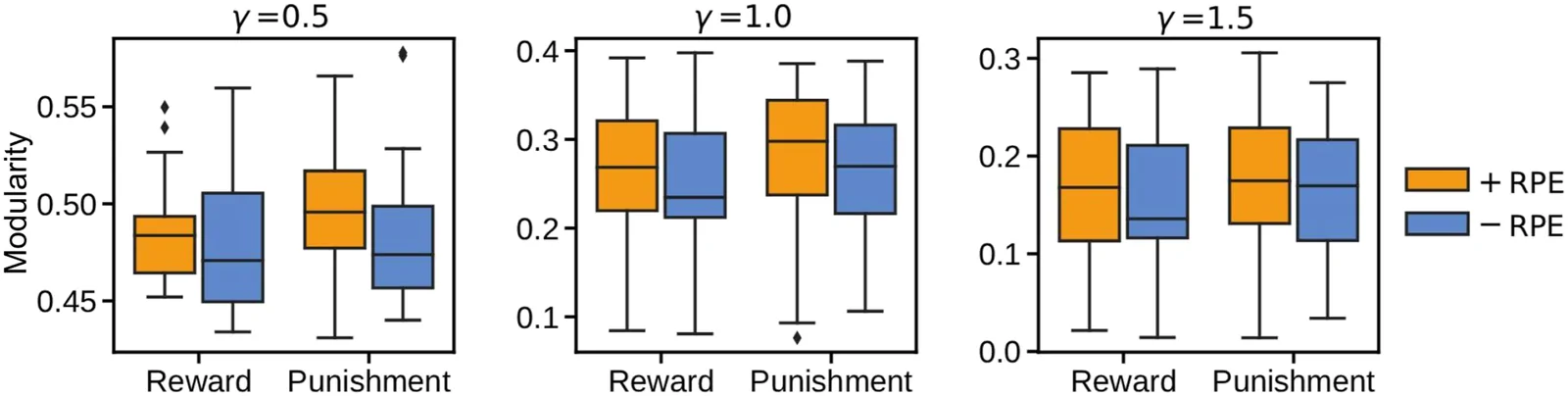
Whole-brain network modularity. Network modularity values for different topological scales and task contexts. Each plot corresponds to a different topological scale characterized by structural resolution parameter *γ*. Modularity values are grouped according to the RPE sign (orange bars = positive RPEs; blue bars = negative RPEs) and task contexts (Reward = reward-seeking context; Punishment = punishment-avoiding context). The boxes show the quartiles of the modularity distribution, while the error bars show the upper and lower limits.

Then, we investigated the modularity of the functional network across three topological scales controlled by *γ* resolution parameters (see the Methods section and Supporting Information for details). On the level of whole-brain network topology, we were interested in whether the overall degree of modularity changes when subjects process positive and negative RPEs in either a risk-seeking or a punishment-avoiding environment. We performed two-way rmANOVA on modularity values with these two factors. The same statistical testing was applied to all three topological scales. The average network modularity was highest for the largest topological scale, *Q*(*γ* = 0.5) = 0.48 ± 0.03, and smallest for the finest topological scale, *Q*(*γ* = 1.5) = 0.16 ± 0.07. It increased significantly with increasing structural resolution parameter (*p* < 10^−10^ paired *t* tests between scales). However, the rmANOVA results were not significant. Neither the main RPE sign effect, the main task-context effect, nor the (RPE sign × task context) effect reached the significance threshold, regardless of the topological scale (Figure 3). This shows that the degree of the whole-brain network segregation expressed as modularity is stable across task contexts and topological scales.

Since a same degree of segregation can characterize a large diversity of network architectures, it is important to analyze networks on finer organizational scales of communities and individual nodes as well. Here, we were interested in whether LSN organization differs between reward-seeking and punishment-avoiding contexts and positive and negative RPEs on different topological scales. We calculated consensus network partitions for each experimental context and topological scale. We also calculated context-independent consensus partitions representing a stable community structure during RPE processing. Context-independent partitions were further used as reference partitions to test context-specific changes in LSN agreement.

As expected, the number of communities increased, and the average community size for context-specific consensus partitions decreased with an increasing structural resolution parameter *γ*. For *γ* = 0.5, consensus partitions consisted of a few “super-communities” (on average 2.25 communities across contexts). The mean “super-community” size was 119.1 ± 43.1 nodes (in the previously chosen ROIs). For intermediate topological scale (*γ* = 1), consensus partitions for all task contexts consisted of three communities, with the average size of 89.3 ± 13.2 nodes per community. Consensus partitions detected for the finest topological scale (*γ* = 1.5) consisted of 41.2 communities per partition (on average across contexts). This proliferation of observed communities resulted from many singletons, that is, communities with one node. Each partition consisted of 27–37 singletons, which decreased the average community size to 6.5 ± 16.1 nodes per community. A similar number of communities and average community size characterized the context-independent consensus partitions. For the “super-community” scale (*γ* = 0.5), the context-independent consensus partition consisted of two communities with 141 and 127 nodes per community. The intermediate scale (*γ* = 1.0) context-independent consensus partition was composed of three communities of size 100, 94, and 74 nodes per community. In the finest topological scale (*γ* = 1.5), the context-independent consensus partition divided the network into 47 communities (38 singletons) with an average community size of 5.7 ± 15.2 nodes per community.

**Figure 4.**
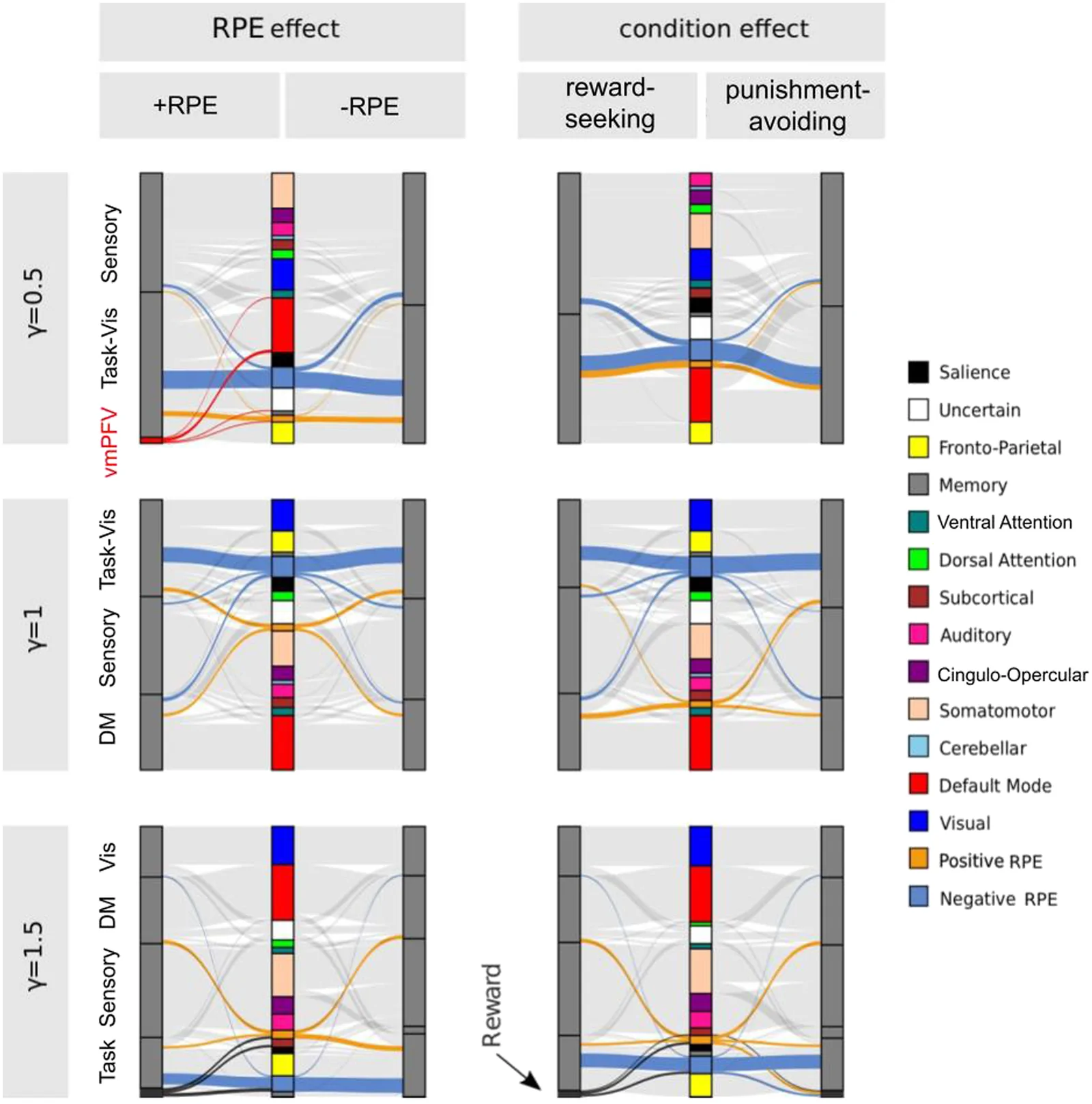
Consensus partitions across topological scales. Sankey diagrams illustrate how task-derived brain communities map onto LSNs. Rows correspond to different topological resolutions (*γ* = 0.5, 1, 1.5), which determine the granularity of detected communities. Columns show different effects: (left-side) separation by reward prediction-error (RPE) sign, (right-side) separation by task context (reward-seeking vs. punishment-avoiding). Colored flows indicate how consensus communities (Task-Vis = task-visual, DM = default mode, Vis = visual) overlap with well-known LSNs (legend, right). Warm-colored bands highlight positive versus negative RPE-related networks. This visualization reveals how community structure shifts with resolution scale, RPE sign, and motivational context.

**Figure 5.**
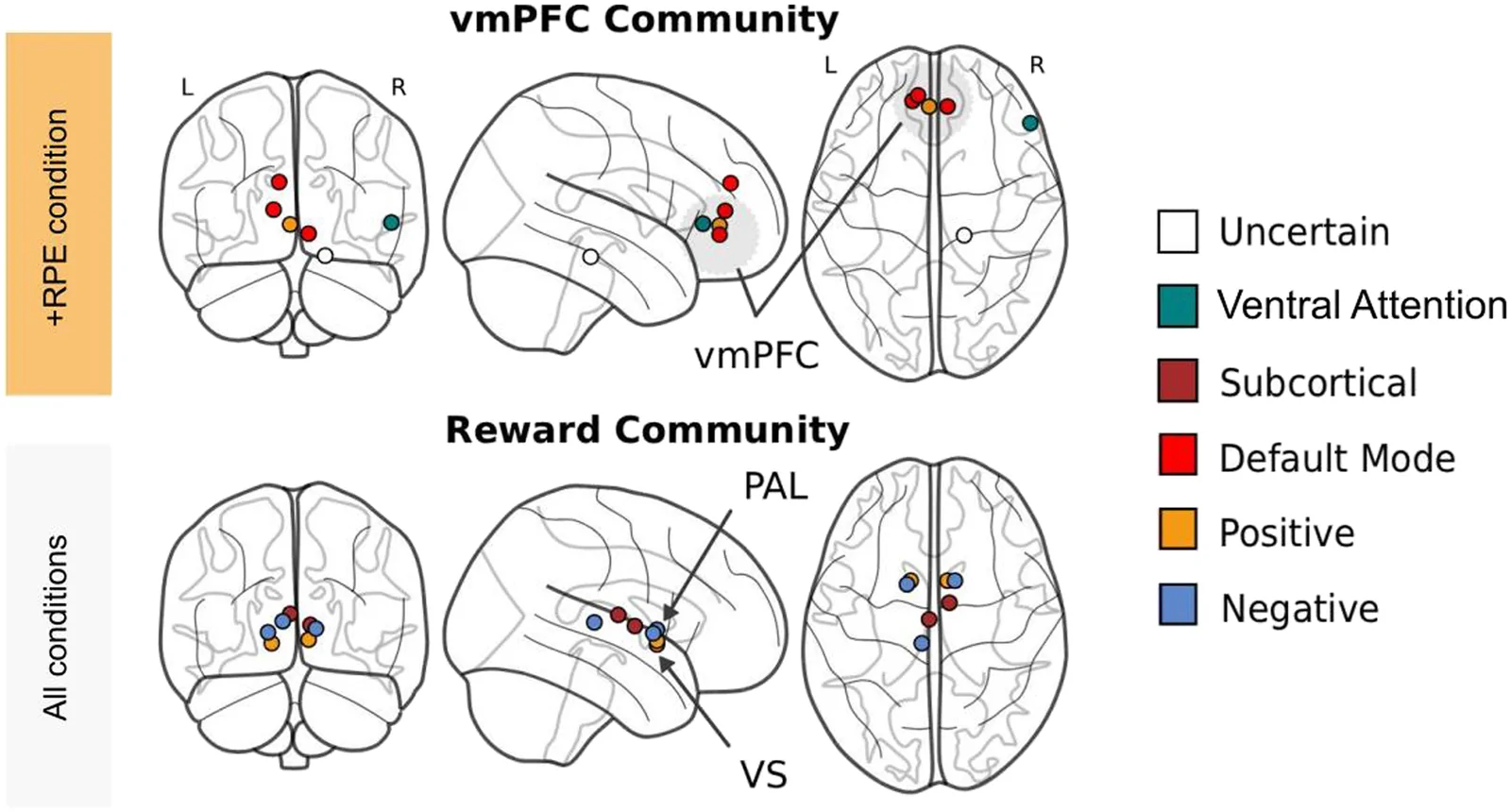
Selected consensus partition communities. (Top panel) The vmPFC community emerging during processing of positive RPEs observed for structural resolution *γ* = 0.5. (Bottom panel) Separate reward community observed for context-invariant consensus partition for structural resolution *γ* = 1.5. It is composed of RPE signaling regions and subcortical network regions and is spatially restricted to the areas around the striatum.

In the coarsest topological scale (*γ* = 0.5), the functional network consisted of two major “super-communities”: the task-visual and the sensory communities (Figure 4, top panel). The task-visual community was composed of task-related networks (RPE signaling, frontoparietal, memory, and salience networks), the DMN, and the visual network. The sensory community consisted of cerebellar, somatomotor, cingulo-opercular, dorsal attention, and auditory networks. Nodes from subcortical and ventral attention networks were divided evenly between the two “super-communities.” Intriguingly, in the reward-seeking context, the visual network was detached from the task-related regions and contributed to the sensory community. Moreover, the positive RPE partition contained a third smaller subnetwork—the vmPFC community—which was not observed for any other context (Figure 5, top panel). Four nodes of this community were located within the vmPFC—an area widely known for representing value information to drive choice (Hare et al., 2009; Rangel et al., 2008). The vmPFC community consisted of six regions: three from the DMN, one from the +RPE network, one from the uncertainty network, and one from the ventral attention network. In the intermediate topological scale (*γ* = 1.0), the pattern of LSNs was similar to the one observed in the “super-community” scale (*γ* = 0.5; Figure 4, middle panel). One notable difference in network organization between these scales was a separation of the DMN and part of the +RPE signaling network from the task-visual community. The default mode community consisted of most DMN nodes (85%–91%), around half of the +RPE signaling, and ventral-attention, as well as uncertainty networks nodes, around 10%–30% subcortical, and 10%–20% of −RPE signaling and frontoparietal network nodes. Interestingly, most −RPE signaling network nodes remained coupled with the task-visual community. In contrast to the coarsest topological scale (*γ* = 0.5), the visual network was consistently coupled with task-related networks for all task contexts.

The analysis of the network partitions for the highest structural resolution (*γ* = 1.5) showed the fine-grained division of the functional network into many smaller communities. Regardless of experimental context, the four largest communities—visual, default mode, sensory, and task—formed the backbone of each consensus partition (Figure 4, bottom panel). Similar to the intermediate scale (*γ* = 1.0), the default mode community consisted of most DMN nodes and regions from +RPE signaling, ventral attention, and uncertainty networks. The task community consisted of regions from frontoparietal, memory, −RPE signaling, +RPE signaling, and salience networks. Interestingly, +RPE signaling regions were split between default mode, task, and reward communities, whereas −RPE signaling regions resided within the task, reward, and other minor communities. In addition to the backbone communities, a small cerebellar community was consistently discovered for all experimental contexts. Moreover, a stable reward community was a part of the consensus partition for +RPE, risk-seeking, and punishment-avoiding contexts. This community was also detected in the context-independent consensus partition representing general network structure during RPE processing. The reward community consisted of five to eight regions from RPE signaling and subcortical networks (see Figure 5, bottom panel). Specifically, the context-independent reward community consisted of bilateral VS and PA, right striatum, and two regions within the thalamus. All regions constituting the reward community were spatially bounded to subcortical areas located around the striatum. The existence of a separate reward network was recently suggested by Huckins et al. (2019) after examining resting-state connectivity patterns in a large cohort of subjects.

### LSN Interactions During RPE Processing

Next, we explored context-specific changes in LSN agreement to test whether modular network architecture fluctuates when subjects process positive and negative RPEs. We were curious if such fluctuations can also be detected when comparing reward-seeking and punishment-avoiding environments. We thus hypothesized that changes related to the RPE sign should be more pronounced than changes related to the task context regardless of the structural resolution.

**Figure 6.**
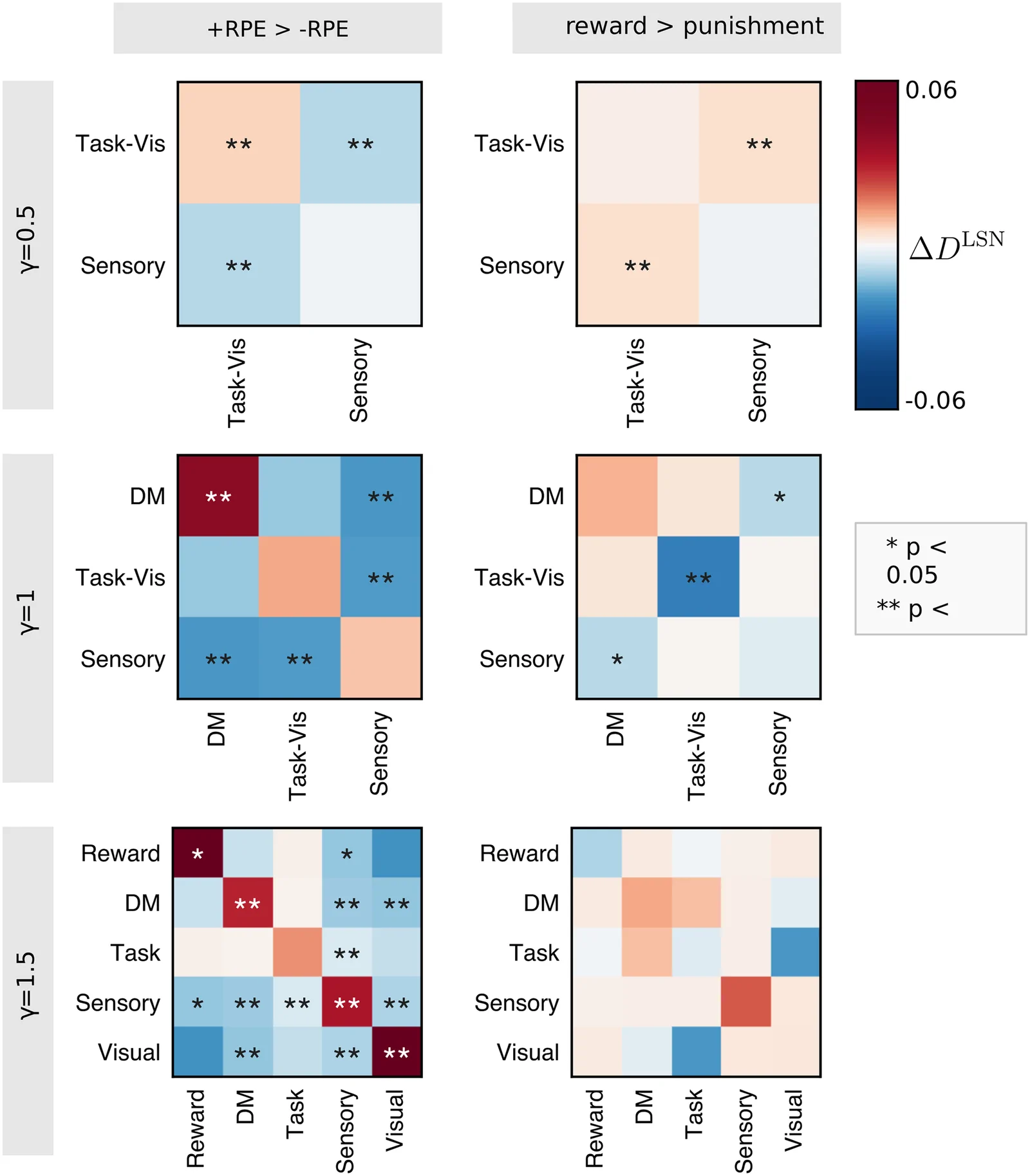
Agreement between LSNs. Changes in within- and between-community agreement for RPE sign and task context. Within-community agreement captures the extent of community segregation, whereas between-community agreement quantifies integration between communities. The left column shows the difference between the agreement during +RPE and −RPE processing. The right column corresponds to the changes between reward-seeking and punishment-avoiding contexts. Rows correspond to topological scales. Reference communities come from context-independent consensus partitions. LSNs abbreviations: Task-Vis = task-visual; DM = default mode.

Functional networks in the coarsest topological scale (*γ* = 0.5) consisted of task-visual and sensory “super-communities” (Figure 6, top panel). Agreement (D) between these “super-communities” was higher during processing −RPEs compared with +RPEs (D_+RPE_ = 0.560, D_−RPE_ = 0.579, *t* = −0.63, *p*_FDR_ [false discovery rate] < 0.0001) and higher in the reward-seeking compared with the punishment-avoiding context (D_RS_ = 0.573, D_PA_ = 0.563, *t* = 0.22, *p*_FDR_ < 0.0001). A significant change of within-community agreement was observed only for RPE sign dimension (no effect detected for the task condition). The task-visual community was more segregated during the processing of +RPEs as indicated by an increased within-community agreement (D_+RPE_ = 0.712, D_−RPE_ = 0.698, *t* = 0.65, *p*_FDR_ = 0.006).

The intermediate topological scale (*γ* = 1.0) was characterized by three LSNs discovered within the consensus partition: default mode, task visual, and sensory (Figure 6, middle panel). Three out of six possible changes in the LSN agreement were significant for the RPE sign dimension. Agreement within the DMN increased during processing +RPEs compared with −RPEs (D_+RPE_ = 0.576, D_−RPE_ = 0.522, *t* = 3.34, *p*_FDR_ < 0.0001). Additionally, the sensory community displayed decreased agreement during +RPEs processing with both default-mode (D_+RPE_ = 0.183, D_−RPE_ = 0.217, *t* = −2.66, *p*_FDR_ < 0.0001) and task-visual communities (D_+RPE_ = 0.214, D_−RPE_ = 0.248, *t* = −3.44, *p*_FDR_ < 0.0001). Note that the between-task decrease in integration for visual and sensory networks was significant for both “super-communities” (*γ* = 0.5) and intermediate scales (*γ* = 1.0), whereas increased segregation of task-visual community was confined only to the coarsest topological scale (*γ* = 0.5). In contrast to the RPE sign effect, only one task-context change remained significant after correction for multiple comparisons. Agreement within the task-visual community was higher in punishment-avoiding compared with the reward-seeking context (D_RS_ = 0.514, D_PA_ = 0.555, *t* = −2.19, *p*_FDR_ < 0.0001). Interestingly, a similar effect was not observed for the coarsest topological scale (*γ* = 0.5). Increased agreement between default-mode and sensory communities during punishment-avoiding context was initially significant but did not survive multiple comparison correction (D_RS_ = 0.192, D_PA_ = 0.208, *t* = −1.11, *p*_unc_ = 0.04).

The consensus partition for the fine-grained topological scale (*γ* = 1.5) consisted of nine (nonsingleton) communities (Figure 6, bottom panel). However, only five of these nonsingletons comprised more than three network nodes. Since minuscule communities are likely byproducts of a reclustering algorithm without significant biological meaning, they were excluded from further LSN agreement analysis. The remaining communities were labeled as reward, default mode, task, sensory, and visual. None of the 15 possible LSN agreement changes between reward-seeking and punishment-avoiding contexts were significant. Contrary, more than half of RPE-sign-related changes turned out to be significant. First, the reward community increased its segregation and decreased its integration with the sensory community with increasing RPE. However, none of these changes survived a multiple comparison correction. Second, the default mode community increased its segregation (D_+RPE_ = 0.549, D_−RPE_ = 0.503, *t* = 2.04, *p*_FDR_ < 0.05) and decreased its integration with sensory (D_+RPE_ = 0.061, D_−RPE_ = 0.082, *t* = −3.59, *p*_FDR_ < 0.0001) and visual communities (D_+RPE_ = 0.037, D_−RPE_ = 0.061, *t* = −3.31, *p*_FDR_ < 0.0001) when switching from negative to positive RPEs processing. Third, the task community had a lower agreement with the sensory community during +RPE processing (D_+RPE_ = 0.047, D_−RPE_ = 0.056, *t* = −3.54, *p*_FDR_ < 0.0001). Fourth, the sensory community increased within-community agreement with increasing RPE (D_+RPE_ = 0.483, D_−RPE_ = 0.434, *t* = 2.31, *p*_FDR_ < 0.0001) and decreased its integration with all remaining communities. Fifth, the sensory community increased within-community agreement with increasing RPE (D_+RPE_ = 0.483, D_−RPE_ = 0.434, *t* = 2.31, *p*_FDR_ < 0.0001) and decreased its integration with all remaining communities. Fifth, the visual community increased segregation during +RPE processing (D_+RPE_ = 0.530, D_−RPE_ = 0.441, *t* = 3.41, *p*_FDR_ < 0.001) and decreased integration with default mode and sensory communities.

Almost all changes related to the RPE sign switching followed the hypothesis of increased within-community agreement and decreased between-community agreement. The only exceptions to that observation were an increased agreement during +RPE processing between the task community and reward/default mode communities for *γ* = 1.5 and a decreased task-visual community agreement during +RPE processing for *γ* = 0.5. However, none of these changes reached the significance level, while all significant effects followed the hypothesis. Interestingly, the community separation effect reflected by increased segregation and decreased integration with other communities was more pronounced for the sensory, default-mode, and visual communities and less pronounced for the task and reward networks.

The RPE sign effects were more evident than the task-context effects for all topological scales, as shown by more significant changes observed for the RPE sign dimension, regardless of the topological scale. This observation was most apparent for the fine-grained topological scale (*γ* = 1.5), where none of the task-context effects reached significance in contrast to nine significant effects for the RPE sign dimension. Moreover, almost all changes related to switching between positive and negative RPEs were scale invariant and spanned two or three scales. For example, a decreased agreement between sensory and task/task-visual communities during +RPE processing was significant for all three scales. In contrast, two significant task-context-related changes were specific to only one single scale. Concretely, a decreased task-visual community agreement during the reward-seeking context was specific to the intermediate scale (*γ* = 1.0), and an increased agreement between the sensory and task-visual communities was significant only for the coarsest scale (*γ* = 0.5).

## DISCUSSION

The overarching goal of our study was to investigate the functional brain network changes during RPE processing in the reward-seeking and punishment-avoiding contexts. On the behavioral level, we found that learning speed depends only on the sign of the RPE and not on the outcome valence. Our behavioral results show that a model with different learning rates for the RPE’s sign but no differentiation based on context came out on top, indicating that participants learned differently for positive and negative surprises, but that the reward context did not have an effect on general learning speed.

Whole-brain network analysis did not show significant differences in network modularity between the processing of positive and negative RPEs. Furthermore, functional networks revealed a multiscale community structure with a separate reward network emerging at a finer topological scale (*γ* = 1.5). Last, the agreement between detected LSNs changed between positive and negative RPE processing. These changes were characterized by a decreased within-network segregation and an increased between-community integration during negative compared with positive RPE processing. This pattern of changes was observed mainly for the default mode, sensory, and visual networks.

### Negative RPEs Processing

From a computational perspective, negative RPEs are simply negative scalar values located on the same scale as positive RPEs. This simple observation raises the question: How does the brain represent negative RPEs? A classical answer is that the dopamine neurons decrease their firing rates, down from baseline rates (Schultz et al., 1997), reducing synaptic dopamine levels in their projective fields. Another possible answer to this question takes the form of the dual systems hypothesis stating that negative RPEs are signaled by a separate neural system outside the dopaminergic circuit (Fouragnan et al., 2018). Our network perspective revealed differential community membership profiles of both systems with the positive RPE system being linked with the default mode network and the negative RPE system being coupled with the frontoparietal network. Behavioral data analysis also supported the duality of RPE signaling—the learning rate depended on the sign of the RPE. A recent fMRI meta-analysis supported these observations by showing a widespread network of areas signaling negative RPEs encompassing the dorsomedial cingulate cortex, anterior insula, PA, and middle frontal gyrus (Fouragnan et al., 2018). A separate community of reward-related regions (nucleus accumbens, lateral and medial orbital frontal cortex, and vmPFC) was recently recognized as a stable LSN at rest (Huckins et al., 2019). This finding suggests that RPE processing regions should be functionally coupled. Based on that, we expected that the distinction between the regions signaling positive and negative RPEs would be reflected by the separate community memberships of these networks.

Here, we analyzed the community structure on three different topological scales. On the coarsest scale (*γ* = 0.5), we found that both RPE systems were a part of a larger task-visual module. Both intermediate (*γ* = 1.0) and fine-grained (*γ* = 1.5) topological scales revealed distinct community membership profiles of both the positive and negative RPE processing regions. On the intermediate scale (*γ* = 1.0), positive RPE regions split evenly between the task-visual and default mode modules, whereas negative RPE regions retained their association with the task-visual module. The fine-grained scale (*γ* = 1.5) revealed even more complex structural patterns. We found a separate reward module composed mainly of striatal regions belonging to both RPE networks. The rest of the RPE signaling regions were divided between the default mode and task modules. The default mode module contained almost half of the positive RPE regions and no negative RPE regions. On the other hand, the task module consisted of both positive and negative RPE regions. These findings partially corroborate the dual system hypothesis (Fouragnan et al., 2018) by showing strong associations between the dopaminergic system and the DMN and the insular system and the fronto-parietal network. Interestingly, these associations indicate that the opposition between the positive and negative RPE systems may be related to the antagonism between a task-negative DMN and a task-positive FPN. Our results show a much more complicated picture of the network community structure in the context of reward-related regions. Most notably, the existence of a separate striatal network comprising both positive and negative RPE processing regions suggests integrated information between the opposing systems. We have to note that contrary to the initial hypothesis, we did not find pure modules composed of positive or negative RPE processing regions. There are two possible explanations—either some of these regions are irreducibly functionally connected or the distinction between them exists, but a finer topological scale is needed to uncover them.

Many studies showed that temporal-difference learning models with asymmetric value updates for positive and negative RPEs are better at explaining learning in humans (Frank et al., 2007; Gershman, 2015, 2016). These models were introduced to take into account the observations supporting the dual systems hypothesis (Frank et al., 2007). If separate neural circuits signal both types of RPEs, their influence on choice value should be independent. On the behavioral level, this influence is operationalized as a scalar value—the learning rate. We hypothesized that models with separate learning rates for positive and negative RPEs should outperform single learning rate models. In line with our prediction and existing literature, we found moderate evidence in favor of this hypothesis. Moreover, the single most frequent model was the model with the RPE-dependent learning rates. This finding further suggests a separation between positive and negative RPE systems on the behavioral level.

### Brain Systems Are Organized Along the RPE Sign Axis

The reference effect hypothesis states that values are not absolute but are actively constructed based on our previous experience and expectations. We can feel positive emotions in generally negative situations and negative emotions when something good happens. For example, when our car breaks down but repair cost is low, we feel relief, or when our boss praises us but our colleagues get a raise, we feel envy and frustration. The reference effect hypothesis offers a solution to the ongoing debate on the primary organizational axis of the brain systems. If values were constructed in absolute terms, brain responses would be organized along the outcome valence axis. However, if values are relative to the experienced context, then brain responses should only reflect the RPE sign axis because prediction mirrors the outcome modulated by expectations acting as a reference. This is relevant to whether brain systems are invariant to the outcome valence and RPE sign on both the behavioral and connectivity level. On the behavioral level, we found that learning rates are invariant to the outcome valence but not to the RPE sign. On the connectivity level, we found that the RPE sign effect sizes are much stronger than the outcome valence effect sizes.

The organization of LSNs constantly changes to meet the demands of the environment (Shine et al., 2016). According to the reference effect hypothesis, the functioning of these systems should be invariant to the outcome valence. From the connectivity perspective, this should imply that changes in modular network structure should be predominately associated with switching between the positive and negative RPEs and not between the reward-seeking and punishment-avoiding contexts. In agreement with this assumption, we found more significant differences for the change in the RPE sign than the change in the outcome valence regardless of the topological scale. Specifically, we found seven significant RPE sign effects for the finer topological scale (*γ* = 1.5) compared with no significant task-context effects. Moreover, switching between different RPE signs was associated with a topologically stable pattern of LSN reorganization. In contrast, outcome valence effects were less consistent and limited to the two coarsest topological scales (*γ* = 0.5 and *γ* = 1.0). Similar to the observed brain activations, these findings support a relaxed version of the reference effect hypothesis—the RPE axis is a dominant organizational axis for brain systems except for a few subtle valence effects.

### Negative RPEs Elicit Brain-Wide Network Reconfiguration

In a reversal learning task, negative RPEs signal either expected random fluctuations or unexpected changes in reward contingencies. Therefore, negative RPE processing may be associated with increased attention and cognitive effort. According to previous studies and theoretical consideration, performing a demanding cognitive task requires long-distance connections integrating specialized neural subsystems—the so-called *global neuronal workspace* (Dehaene et al., 1998). On the other hand, the brain can perform less cognitively demanding tasks within a set of functionally specialized modules. From the network science perspective, these two effects correspond to an increased within-community integration and a decreased modularity, which signals a decreased between-community segregation. Several studies reported these changes in functional brain networks during cognition (Finc et al., 2017; Shine et al., 2016; Vatansever et al., 2015). Similar to previous studies, we expected to observe a decreased whole-brain modularity, and thus a decreased within-community segregation, as well as an increased between-community integration when switching from positive to negative RPE processing. In line with these expectations, we observed decreased segregation of the default mode, sensory and visual modules, and increased integration between the default mode and sensory/visual modules during negative RPE processing. However, overall network modularity was stable across both RPE signs and task contexts.

Although the whole-brain modularity values were lower for negative RPEs in all three topological scales, none of these values were significant. There are two possible explanations for that observation. First, in the case of a probabilistic learning task, cognitive effort differences between positive and negative RPEs could be much lower than similar differences during working memory or attention tasks. Subtle differences in cognitive effort would lead to a smaller modularity breakdown that cannot be detected with the sample size used in our study. This explanation is consistent with the observation of a lower modularity for negative RPEs in all topological scales. Second, the reorganization effect may be specific to changes in the composition of network communities without altering the macroscopic level of global modularity (Sporns, 2014). Similar to our results, a recent study on number comparison reported significant alterations of network community structure without changes in whole-brain network modularity (Conrad et al., 2020).

On the coarsest topological scale (*γ* = 0.5), the DMN was a part of the task-visual module. This module presented with decreased segregation and increased integration with the sensory module during negative RPE processing. In the intermediate (*γ* = 1.0) and finest (*γ* = 1.5) topological scales, DMN formed a separate module. Similar to the task-visual module, this module presented with decreased segregation and increased integration with the sensory and visual modules. Recent work demonstrated that the DMN may play a crucial role in the formation of an integrated workspace (Finc et al., 2017). In the context of RL, DMN contains a part of the RPE signaling network, most notably the vmPFC commonly associated with value representation (Frank & Claus, 2006; Rangel et al., 2008). Our findings suggest that DMN reorganization patterns may reflect (a) global workspace formation and (b) enhanced communication between the valuation circuit and other brain systems following negative RPE processing.

The sensory network contains the motor cortex responsible for the movement execution following the subject’s decision. Some studies suggest that motor regions may implement a winner-take-all mechanism integrating values of different stimuli (Cisek & Kalaska, 2005). Moreover, Horga et al. (2015) demonstrated the importance of sensorimotor connectivity for RL during gradual learning in a virtual maze. Interestingly, our results show that the sensory network increases its agreement during negative RPE processing only with all other systems regardless of the scale. This suggests a potential need for information integration between the choice and valuation circuit and other LSNs.

We observed the separate reward network only for finer topological scale (*γ* = 1.5). The reward network consisted of a few striatal regions signaling both types of RPEs. Consistently with the reconfiguration pattern of the global workspace formation, the reward network presented with decreased segregation and increased integration with the sensory network during negative RPE processing. Decreased segregation of striatal regions may reflect functional decoupling of positive and negative RPE processing regions during a more effortful type of RPE processing. This decoupling possibly allows reducing interference between antagonistic areas when more cognitive resources are required. We have to note that reward system effects did not survive multiple comparison corrections and thus need to be interpreted with caution. A smaller effect size for the reward network may be related to a smaller community size of only seven nodes.

Agreement analysis revealed only two changes in LSN integration and segregation for the outcome valence axis. Both changes were related to the task-visual module and were observed for the coarse (*γ* = 0.5) and intermediate (*γ* = 1.0) topological scales. This module had increased integration and decreased segregation with the sensory module during the reward-seeking context. One possible explanation of this effect is the influence of systematic differences in stimulus presentation during the outcome phase described in the previous section. In the activation analysis, we observed differences in RPE processing in a large portion of the primary and secondary visual cortices, a part of the task-visual module. It is also important to note that despite statistical significance, both effects were scale specific and were not present for other topological resolutions, unlike RPE-related changes.

### Ventromedial Prefrontal Regions Form a Separate Network During Positive RPE Processing

The vmPFC is part of one of the main subsystems of the DMN (Andrews-Hanna et al., 2014). From the RL perspective, the vmPFC signals positive RPEs (Daw et al., 2011; van den Bos et al., 2012) and represents a subjective value of various stimuli (Levy & Glimcher, 2012; Roy et al., 2012). Using consensus partitioning, we found that regions spanning the vmPFC area formed a separate network community during positive RPE processing.

Surprisingly, this community was present only for the coarsest topological scale (*γ* = 0.5), which inherently favors larger “super-communities.” This may suggest remarkably strong connections among vmPFC nodes during positive RPE processing. It has been previously suggested that value representation in vmPFC is updated by striatal RPEs through strong frontostriatal connections (Frank & Claus, 2006). Connectivity studies supported this claim by showing increased functional coupling between the VS and the vmPFC during feedback processing (Camara et al., 2009; Münte et al., 2008). Moreover, van den Bos et al. (2012) showed that connection strength between these two regions is enhanced during positive RPE processing. This value update mechanism offers a possible explanation for our finding—the dopaminergic system may communicate positive RPEs with the vmPFC through frontostriatal connections evoking strong and coherent neural activity in different parts of the vmPFC. Strong coherent activity results in strong functional coupling, which results in a separate vmPFC community observed during positive RPE processing. The other complementary mechanism for community formation is decreased connectivity with noncommunity members. From the RPE processing perspective, this would reflect the need for the valuation circuit in vmPFC to reduce noise from other brain systems during value updating. Only one or both of the suggested mechanisms may be responsible for the vmPFC community formation. Therefore more studies and fine-grained network structure analysis would be needed to understand this phenomenon better. Since this result was unexpected, it should be treated as exploratory and interpreted with caution.

While our interpretation is based on group-level analyses (*N* = 29), future studies should examine the robustness of community-level characterizations across individuals. Cross-validation approaches could help assess the consistency of ROI-to-community assignments in the 2 × 2 comparison and evaluate the generalizability of observed network effects. Furthermore, the robustness of community detection across task conditions could be affected by degeneracy issues inherent to modularity optimization. Although we employed consensus clustering to reduce variability across Louvain runs, future analyses should systematically test the robustness of ROI community assignments across multiple iterations before consensus formation—particularly at higher *γ*-values, where smaller communities may be more susceptible to noise and resolution-limit effects.

While several findings were consistent with our predictions, some results did not align with the initial hypotheses, for example, the much more complicated network community structure in the context of reward-related regions, a stable network modularity across RPE signs and task contexts, and the separate network community during positive RPE processing spanning the vmPFC area only for the coarsest topological scale (*γ* = 0.5).

### Conclusions

Multiple neural systems are engaged to signal RPEs enabling us to learn through trial and error. The results of our study show that these systems are mainly organized along the RPE sign axis, reflecting the distinction between positive and negative RPEs. Moreover, the activity and connectivity of these systems are generally invariant to the outcome valence, supporting the idea that the brain adjusts its reference point when the environment is predominantly rewarding or punishing. Our results also demonstrate a complex pattern of network interactions following switching between positive and negative RPEs. These interactions form the pattern observed in studies on cognitive load, suggesting that negative RPEs require more cognitive resources as they are usually related to the conflict. Intriguingly, we also found some unexpected network community structures. Ventromedial prefrontal regions formed a small community observed along two large “super-communities,” suggesting increased integrity of the valuation system and decreased communication with the rest of the brain during positive RPE processing. Striatal areas composed of both regions signaling positive and negative RPEs formed a stable reward network observed for higher topological resolutions supporting the finding of a separate reward network observed at rest. Our findings provide a thorough description of the neural correlates of RPEs from three different perspectives. They support and extend some existing observations and shed new light on the network mechanisms behind RL. Some of our results raise new fascinating questions and open avenues for future research.

## METHODS

### Participants

Thirty-two healthy volunteers (14 female; mean age: 20.9 ± 2.24; age range: 18–28) were recruited from the local community through social networks and word of mouth. All participants were right-handed, had a normal or corrected-to-normal vision, and did not suffer from neurological or psychiatric disorders at the time of examination or in the past. Informed consent was obtained in writing from each participant, and ethical approval for the study was obtained from the Ethics Committee of the Nicolaus Copernicus University Ludwik Rydygier Collegium Medicum in Bydgoszcz, Poland.

Please note that for the neural analyses, three participants were excluded due to excessive motion artifacts, based on predefined exclusion criteria: a mean framewise displacement (FD) greater than 0.2 mm in at least one condition, a maximum FD exceeding 5 mm in any condition, or more than 20% of volumes with FD > 0.5 mm in any condition. These criteria ensured high-quality data and minimized motion-related artifacts in the imaging analyses. For the behavioral analyses, we did keep these participants, and thus a total *n* of 32 was reported.

### Experimental Procedures

A PRL task was selected to examine the neural correlates of RPEs (Cools et al., 2002). The PRL task consisted of two contexts, reward-seeking and punishment-avoiding. In both contexts, winning and losing are opposed to neutral outcomes, enabling disentangling between often confused dimensions: the outcome valence and the RPE sign (Palminteri & Pessiglione, 2017). Both task contexts were performed inside the fMRI scanner. Participants were instructed to repeatedly choose between yellow and blue boxes to collect as many points as possible in the reward-seeking context or lose as few points as possible in the punishment-avoiding context (Figure 1A). One of the boxes had the probability of being correct (rewarding or nonpunishing depending on the context) *p* = 0.8 and the other one *p* = 0.2. These probabilities were unknown to the subjects and had to be learned from experience. The reward/punishment contingency changed four times throughout each task context (Figure 1C). Each box had an associated reward/punishment magnitude, randomly selected at the beginning of each trial. Magnitudes for both boxes were integers summing up to 50, with the difference between them not exceeding 40. They were represented as white numbers on the boxes, indicating possible gain in the reward-seeking context or loss in the punishment-avoiding context. Successful performance in the PRL task requires the decision-maker to correctly estimate accurate choice probabilities from experience and integrate them with reward/punishment magnitudes to choose an option with a higher expected value.

Each task context had a separate fMRI run and consisted of 110 trials. Each trial began with the decision phase indicated by the question mark appearing within the fixation circle (Figure 1B). During the decision phase, a subject had 1.5 s to choose one of the boxes by pressing a button on the response grip with either the left or right thumb. The decision phase was followed by a variable interstimulus interval (ISI; 3–7 s, jittered), after which an outcome was presented for 1.5 s. During the outcome phase, the fixation circle was colored according to the rewarded or punished box, and the number within the circle represented the number of gained or lost points. The outcome phase was followed by a variable intertrial interval (ITI; 3–7 s, jittered).

The gray account bar at the bottom of the screen showed points that the subject gathered in the reward-seeking context or the remaining points in the punishment-avoiding context. In the reward-seeking context, subjects were informed that if they fill half of the bar or the entire bar, they will receive 10 PLN (≈ 2.5 USD) or 20 PLN (≈ 5 USD), respectively. Similarly, in the punishment-avoiding context, they were informed that they would receive 20 PLN if left with more than half of the bar, 10 PLN if left with less than half of the bar, and no money if they lose all of their points. To maintain a constant motivation throughout the task, incentive thresholds were set such that all participants acquired at least 10 PLN from either task.

During the task, participants were explicitly instructed that one of two boxes (without telling them which) will be more often rewarded in the reward-seeking context or more often punished in the punishment-avoiding context and that this contingency may reverse several times throughout the task. Before the MRI scan, subjects practiced both task contexts on the lab computer. During the first phase of the practice, participants were provided with feedback indicating which box is more frequently correct to ensure that they grasp the correct model of the task environment. This is done because it was found that heterogeneity in participants’ prior expectations regarding the task structure may lead to heterogeneity in behavior even in simple tasks leading to inaccurate behavioral modeling (Shteingart & Loewenstein, 2014).

The tasks were presented on the MRI compatible NNL goggles (NordicNeuroLab, Bergen, Norway) using PsychoPy software (v. 1.90.1; www.psychopy.org; Peirce, 2007). The behavioral responses were collected using MRI-compatible NNL response grips (NordicNeuroLab, Bergen, Norway), which were held in both hands. Each task lasted approximately 24 min. The order of the task contexts and the colors for the left and right boxes (yellow and blue) were counterbalanced across subjects.

### The Behavioral Model Space

RL models can quantitatively account for learning by trial and error (Montague et al., 1996). Precisely, models based on the idea of the temporal-difference learning postulate that stimulus values are updated proportionally to the RPE and weighted by an adjustable learning rate (Rescorla & Wagner, 1972). Because of the anticorrelated design of correct choice probabilities during the PRL task, simultaneous stimulus value updates for the chosen and nonchosen options were assumed (O’Doherty et al., 2007). Following previous studies, models with separate learning rates for positive and negative RPEs were used to account for possible risk-sensitivity effects. These models would be referred to as prediction-error-dependent models (Niv et al., 2012; Reiter et al., 2016; van den Bos et al., 2012). To capture possible valence effects, for example, loss aversion, models with separate learning rates for reward-seeking and punishment-avoiding contexts were also included. These models would be referred to as context-dependent models. To fully explore the model space, all possible combinations of models were considered, which resulted in four different models:

1. PICI model, with a single learning rate independent of the RPE sign or the outcome valence
2. PICD model, with two separate learning rates for the reward-seeking and punishment-avoiding contexts
3. PDCI model, with two separate learning rates for the positive and negative RPEs
4. PDCD model, with four separate learning rates for the RPE signs and the outcome valence

All models assumed that the RPE at each trial *t* is computed as the difference between the experienced outcome (*r*_*t*_) and the expected probability that the chosen option will be correct (rewarding / not-punishing), $p_{t}^{c}$:

$$\delta_{t}=r_{t}-p_{t}^{c}$$ (1)

Outcomes could be either *r*_*t*_ = 1 when a stimulus is rewarded / not punished or *r*_*t*_ = 0 when a stimulus is punished / not rewarded at trial *t*. Note that the randomly drawn reward magnitudes did not follow any structured rules. Hence, participants learned and estimated only correct choice probabilities and not action values. In each trial, probability estimates for both the chosen, $p_{t}^{c}$, and the unchosen stimulus, $p_{t}^{u}$, were simultaneously updated according to a standard Rescorla–Wagner rule:

$$\left\{\begin{array}{l} p_{t}^{c}=p_{t-1}^{c}+\alpha_{t}\delta_{t}\\ p_{t}^{u}=p_{t-1}^{u}-\alpha_{t}\delta_{t} \end{array}\right.$$ (2)

where *α*_*t*_ ∈ [0, 1] is the learning rate used in trial *t*. Note that the learning rate values close to zero result in minor updates and slow learning, whereas values close to one result in probability matching behavior. In the simplest PICI model, a single learning rate, $\alpha_{t}^{\text{PICD}}$, was used to update probability estimates regardless of RPE sign and task context. The rest of the models assumed separate learning rates depending on task context, RPE sign, or both:

$$\begin{array}{l} \alpha_{t}^{\text{PICD}}=\left\{\begin{array}{l} \alpha^{\text{RS}}\;\text{if}\;\text{condition}\;\text{is}\;\text{reward-seeking}\\ \alpha^{\text{PA}}\;\text{if}\;\text{condition}\;\text{is}\;\text{punishment-avoiding} \end{array}\right.,\\ \alpha_{t}^{\text{PDCI}}=\left\{\begin{array}{l} \alpha^{+}\;\text{if}\;\delta_{t}>0\\ \alpha^{-}\;\text{if}\;\delta_{t}<0 \end{array}\right.,\\ \alpha_{t}^{\text{PDCD}}=\left\{\begin{array}{l} \alpha^{\text{RS}+}\;\text{if}\;\delta_{t}>0\;\text{and}\;\text{condition}\;\text{is}\;\text{reward-seeking}\\ \alpha^{\text{RS}-}\;\text{if}\;\delta_{t}<0\;\text{and}\;\text{condition}\;\text{is}\;\text{reward-seeking}\\ \alpha^{\text{PA}+}\;\text{if}\;\delta_{t}>0\;\text{and}\;\text{condition}\;\text{is}\;\text{punishment-avoiding}\\ \alpha^{\text{PA}-}\;\text{if}\;\delta_{t}>0\;\text{and}\;\text{condition}\;\text{is}\;\text{punishment-avoiding} \end{array}\right.. \end{array}$$ (3)

Action selection was modeled based on the reward/punishment magnitudes and the continuously updated probability estimates. Utility of both options was assumed as Pascalian expected value, that is, a product of the expected probability that the box is rewarded/punished *ρ*_*t*_, and the reward/punishment magnitude for that box, *x*_*t*_:

$$v_{t}^{\text{left}/\text{right}}=\rho_{t}^{\text{left}/\text{right}}x_{t}^{\text{left}/\text{right}}$$ (4)

Note that in the case of the punishment-avoiding context, the expected probability that the stimulus leads to punishment equals one minus the expected probability that it is a correct choice:

$$\rho_{t}=\left\{\begin{array}{ll} p_{t} & \text{if}\;\text{condition}\;\text{is}\;\text{reward-seeking}\\ 1-p_{t} & \text{if}\;\text{condition}\;\text{is}\;\text{punishment-avoiding} \end{array}\right.$$ (5)

Finally, the choice probability was derived by coupling the expected values with the softmax policy rule (Luce, 1957):

$$\left\{\begin{array}{l} P_{t}^{\text{left}}=\exp^{-1}\left(\beta \left(v_{t}^{\text{left}}-v_{t}^{\text{right}}\right)\right)\\ P_{t}^{\text{right}}=1-P_{t}^{\text{left}} \end{array}\right.$$ (6)

where the precision (or “inverse-temperature”) parameter *β* ∈ [0, ∞) reflects the choice stochasticity by controlling the sensitivity of the choice probability to differences in the expected value between the two stimuli. Choice probability values served as likelihood functions generating choices in the Bayesian modeling framework.

### Behavioral Performance

Successful performance in the PRL task requires subjects to take into account the changing reward or punishment probabilities and magnitudes associated with each choice. As in any version of the PRL task, probabilities had to be learned from experience by trial and error. The individual performance accuracy of every subject was calculated to establish if the subjects succeeded in learning the probabilistic associations. The accuracy was defined as the proportion of choices that led to rewarding outcomes in the reward-seeking context or nonpunishing outcomes in the punishment-avoiding context. Since the study examined healthy subjects without valence-dependent learning deficits, we hypothesized similar performance levels for both task contexts.

### Bayesian Modeling

A Bayesian HLM was used to perform model selection and parameter estimation. This type of Bayesian model contains parameters representing behavioral parameters like learning rates or precision, and parameters reflecting the belief about which competing model generates the observed data. The hierarchical structure of the model assumes that behavioral parameters of individuals come from group-level distributions. The latent-mixture structure assumes that the observed behavior can arise as a combination of computations from different cognitive processes. The HLM was estimated with the PICI, PICD, PDCI, and PDCD submodels in the model comparison (Supporting Information Figure S1). Afterward, a hierarchical latent mixture model with a winning submodel as a generative model for behavioral responses was estimated for parameter estimation purposes.

Weakly informative hyperpriors were set to model the distribution of the learning rates and precision across individuals. Following previous studies, learning rates were assumed to come from a beta distribution (Gershman, 2016). A beta distribution is defined for the continuous interval *α* ∈ [0, 1] covering the entire range of possible learning rate values:

$$f\left(a,b,\alpha \right)=c_{a,b}\alpha^{a-1}{\left(1-\alpha \right)}^{b-1}$$ (7)

where *a* and *b* are two nonnegative shape parameters. These were set to come from the uniform hyperprior distributions *a* ∼ Uniform(1, 10) and *b* ∼ Uniform(1, 10). Note that both shape parameters were assumed greater than 1, as the beta distribution takes a behaviorally implausible U-shape if *a* < 1 and *b* < 1. Precision parameter *β* was independently included for each submodel to provide additional flexibility in the model parameter space and avoid potential dependence between competing models. The generative distribution for the precision parameter *β* was represented as a lognormal distribution. Hyperpriors for the lognormal parameters distributions were set to *μ* ∼ Uniform(−2.3, 3.4) for the lognormal group mean and *σ* ∼ Uniform(0.01, 1.6) for the lognormal group standard deviation. These weakly informative hyperpriors were used in previous studies and derived from the constraints to confine a group mean for the precision parameter within the 0.1 to ∼30 interval (Meder et al., 2019; Nilsson et al., 2011). The latent-mixture part of the model assumed a subject-specific model indicator variable to represent a latent mixture of four competing submodels. This assumption allowed us to model each subjects’ response pattern separately by using a different mixture of competing submodels. The model indicator variable, *z*_*i*_, was modeled with a uniformly distributed flat prior representing a lack of expectations toward which behavioral model best explains the subjects’ response patterns.

The *Markov Chain Monte Carlo* (MCMC) sampling was performed using JAGS software (v4.3.0; https://mcmc-jags.sourceforge.net/) called from MATLAB R2017a via matjags.m script (v1.3.3; https://github.com/msteyvers/matjags). A sampling procedure with 2,000 burn-in samples, four chains, and 15,000 samples per chain was used to estimate the HLM (see Supporting Information). The single best submodel was selected by calculating the estimated model frequencies and protected exceedance probabilities (Rigoux et al., 2014) for the highest model frequency among other submodels. These calculations were performed with the Variational Bayesian Analysis toolbox (v1.9.2; https://github.com/MBB-team/VBA-toolbox), which implements Bayesian model selection for group studies. Model parameters for the winning model were fitted by estimating a reduced HLM.

The HLM with four competing submodels was evaluated to select the winning submodel. A sampling of the probability distribution of the model indicator variable *z*_*i*_ resulted in a posterior distribution for each subject reflecting the posterior probability for each competing submodel.

### Data Acquisition

Brain imaging data were collected using a GE Discovery MR750 3 Tesla fMRI scanner (General Electric Healthcare) with a standard eight-channel head coil. Anatomical images were obtained using a three-dimensional high resolution T1-weighted (T1w) gradient-echo (FSPGR BRAVO) sequence (TR = 8.2 s, TE = 3.2 ms, FOV = 256 mm, flip angle = 12°, matrix size = 256 × 256, voxel size = 1 × 1 × 1 mm, 206 axial oblique slices). Functional images were obtained using a T2*-weighted gradient-echo, echo-planar imaging (EPI) sequence sensitive to BOLD contrast (TR = 2,000 ms, TE = 30 ms, FOV = 192 mm, flip angle = 90°, matrix size = 64 × 64, voxel size 3 × 3 × 3 mm, 0.5-mm gap). Two runs of the PRL task in the reward-seeking and punishment-avoiding contexts were acquired (24 min 20 s; 730 volumes each). Forty-two axial oblique, interleaved slices were scanned for each functional run, and five dummy scans (10 s) were collected at the beginning of each run to stabilize magnetization.

### Data Processing

The raw DICOM data were converted to NifTI format, structured according to the Brain Imaging Data Structure (BIDS) standard, and validated using BIDS Validator (https://bids-standard.github.io/bids-validator/; Gorgolewski et al., 2016). The preprocessing was performed using fMRIPrep version 1.4.1 (Esteban et al., 2019; Gorgolewski et al., 2011; see Supporting Information for the full description of the pipeline).

### Brain Parcellation

To extract brain signals, we used the parcellation introduced by Power et al. (2011) that consists of 264 ROIs, which had been defined based on a meta-analysis of activation patterns from multiple studies. Each ROI is modeled as a 5-mm sphere around the specific MNI coordinates. Power’s parcellation additionally includes the division of the 264 ROIs into 13 LSNs: auditory, cerebellar, somatomotor, cingulo-opercular, default mode, memory, ventral attention, dorsal attention, frontoparietal, salience, subcortical, uncertainty, and visual. This division allows for the examination of the dynamic interplay between the LSNs in various experimental contexts. However, Power’s parcellation does not contain ROIs of distinct RPE-related networks; therefore, it cannot be directly used to study how the reward system interacts with the rest of the brain during an RL task. Here, Power’s ROIs were combined with regions from the recent meta-analysis on neural representations of the RPE sign (Fouragnan et al., 2018; see Supporting Information for details).

### Beta-Series Correlation

The task-related functional connectivity was estimated using a beta-series correlation method introduced by Rissman et al. (2004). The beta-series correlation method quantifies event-to-event fluctuations in the activity of different brain areas. These fluctuations allow for the estimation of statistical dependency between regional activations for different event types. The “single-trial-versus-other-trial” approach introduced by Mumford et al. (2012) was used to compute the activation beta maps. This approach creates a separate GLM for each trial in which the trial of interest is modeled as a separate regressor, and all other trials are modeled as single nuisance regressors. Here, we used the FirstLevelModel class from the Nistats package and a custom code to calculate the beta-series correlation. The GLM consisted of (a) one regressor modeling a trial of interest as an event of duration 0 s (typically used to model very short cognitive processes) time-locked to the onset of the outcome phase, convolved with a standard Statistical Parametric Mapping (SPM) hemodynamic response function (HRF); (b) two regressors modeling trials with positive and negative RPEs as events of duration 0 s time-locked to the onset of the outcome phase, convolved with an HRF; (c) six regressors modeling the events of no interest: decision onset, decision onset modulated with expected probability of being correct, decision onset for missed trials, left button press, right button press, and decision offset for missed trials; (d) 24 head motion parameter regressors modeling motion-related noise; (e) cosine functions modeling low-frequency scanner drift (see Supporting Information for details).

The output of this procedure consisted of a three-dimensional beta map for each trial, indicating the voxel-wise estimate of the neuronal activity evoked during this trial. After GLM estimation, the beta values for each trial were averaged in each of the 264 ROIs, producing 264 beta-series per subject and per task context. The beta-series were then *z* transformed (within conditions) and separated by the RPE sign of each trial. This procedure gave four beta-series for each participant: +RPE trials in the reward-seeking context, −RPE trials in the reward-seeking context, +RPE trials in the punishment-avoiding context, and −RPE trials in the punishment-avoiding context. Beta-series were then correlated using Pearson’s correlation coefficient, generating a *N ROI* × *N ROI* symmetrical correlation matrix for each subject, task context, and trial-wise RPE sign. Correlation values were then converted to *z* scores using Fisher *z* transform. Transformed matrices represented the final subject-level estimate of the functional connectivity pattern evoked by the specific task context and the type of trial (see Supporting Information for details).

### Multiscale Modularity

To calculate community structure properties across topological scales, the structural resolution parameter space was sampled by varying *γ* from 0.05 to 3 in step of 0.05. For each value of *γ*, the Louvain modularity maximization algorithm was used to quantify the optimal network structure (Blondel et al., 2008). Negative connections were separately included in the modularity function and treated asymmetrically as suggested in Rubinov and Sporns (2011):

$$Q^{\text{asym}}\left(\gamma \right)=Q^{+}\left(\gamma \right)+\frac{\upsilon^{-}}{\upsilon^{-}+\upsilon^{+}}Q^{-}\left(\gamma \right)$$ (8)

where *Q* ± (*γ*) are modularity values calculated separately for positive and negative connections and *v* ± = *ij A* ± *ij* are total connection strengths, that is, costs for positive and negative connections. For each functional network, the Louvain algorithm was run 100 times, and the partition with the highest value of *Q asym* (*γ*) was kept for further analysis. Several metrics were computed to inspect different topological scales (Supporting Information Figure S6): (a) a mean number of communities, singletons, and nonsingletons (communities with more than one node), (b) the mean community size, (c) the mean similarity between data-driven partitions, and (d) the mean similarity between the reference partition and the data-driven partitions. The partition similarity was computed as the normalized mutual information between community partition vectors (Meilă, 2007). A sparse sampling of *γ* range was employed, enabling to focus on three distinct topological scales: *γ* = 0.5 representing the largest scale of a few “super-communities” consisting of multiple LSNs, *γ* = 1 representing the most frequently studied intermediate scale, and *γ* = 1.5 representing the finer scale approximately resembling a well-known division into LSNs (see Supporting Information for details).

To assess subject-level modularity and community structure for both task contexts and trial-wise RPE signs, we employed a similar procedure to the one used during the structural resolution parameter analysis. For topological scale and each functional network, the Louvain modularity maximization algorithm was executed 1,000 times, and the output of the run with the highest modularity was saved for further analysis. The output consisted of the weighted modularity with the asymmetric treatment of negative weights, *Q asym* (called *Q* in subsequent analysis), and an *N ROI* × 1 community affiliation vector, *M*, whose *i*th element indexed the community affiliation of node *i*.

### Consensus Partitioning

A consensus partition represents the modular structure of a functional brain network during a specific experimental context. The group-level community structure represented by the consensus partition can be calculated from the subject-level community affiliation vectors. The consensus partitions effectively decrease the data dimensionality, allowing a qualitative description of the network reconfiguration. There are two popular approaches for defining a consensus partition: (a) selecting a partition with the highest similarity to the other partitions (Doron et al., 2012) or (b) reclustering an association matrix that stores the information about pairwise nodal co-occurrence within the same community (Bassett et al., 2013; Betzel et al., 2017; Lancichinetti & Fortunato, 2012). Here, the latter approach based on the concept of agreement (or module allegiance) was used. The agreement, *D*_*ij*_, is a measure defined for a pair of nodes, *i* and *j*, and characterizes the extent to which these nodes belong to the same community (Bassett et al., 2015; Bertolero et al., 2015):

$$D_{ij}=\frac{1}{N}\sum_{k=1}^{N}a_{ij}^{k}$$ (9)

where *N* is the number of partitions. *K*_*ij*_ equals 1 if the nodes *i* and *j* belong to the same community, and 0 otherwise. The elements of the agreement matrix *D*_*ij*_ reflect the probability that nodes *i* and *j* are found within the same community for a randomly selected partition given a set of partitions. Although the information represented by the agreement matrix is not independent of the information contained in the connectivity matrix, an agreement can provide a complementary characteristic of nodal associations. For example, two nodes can share a weak direct connection but many strong indirect connections. In this case, there is a high chance that they will be placed within the same community exhibiting both significant agreement and low connectivity at the same time. Furthermore, Bassett et al. (2015) has shown that agreement reduces the noise in connectivity matrices and is more sensitive to differences in the network organization compared with information contained in the connectivity values alone.

**Figure 7.**
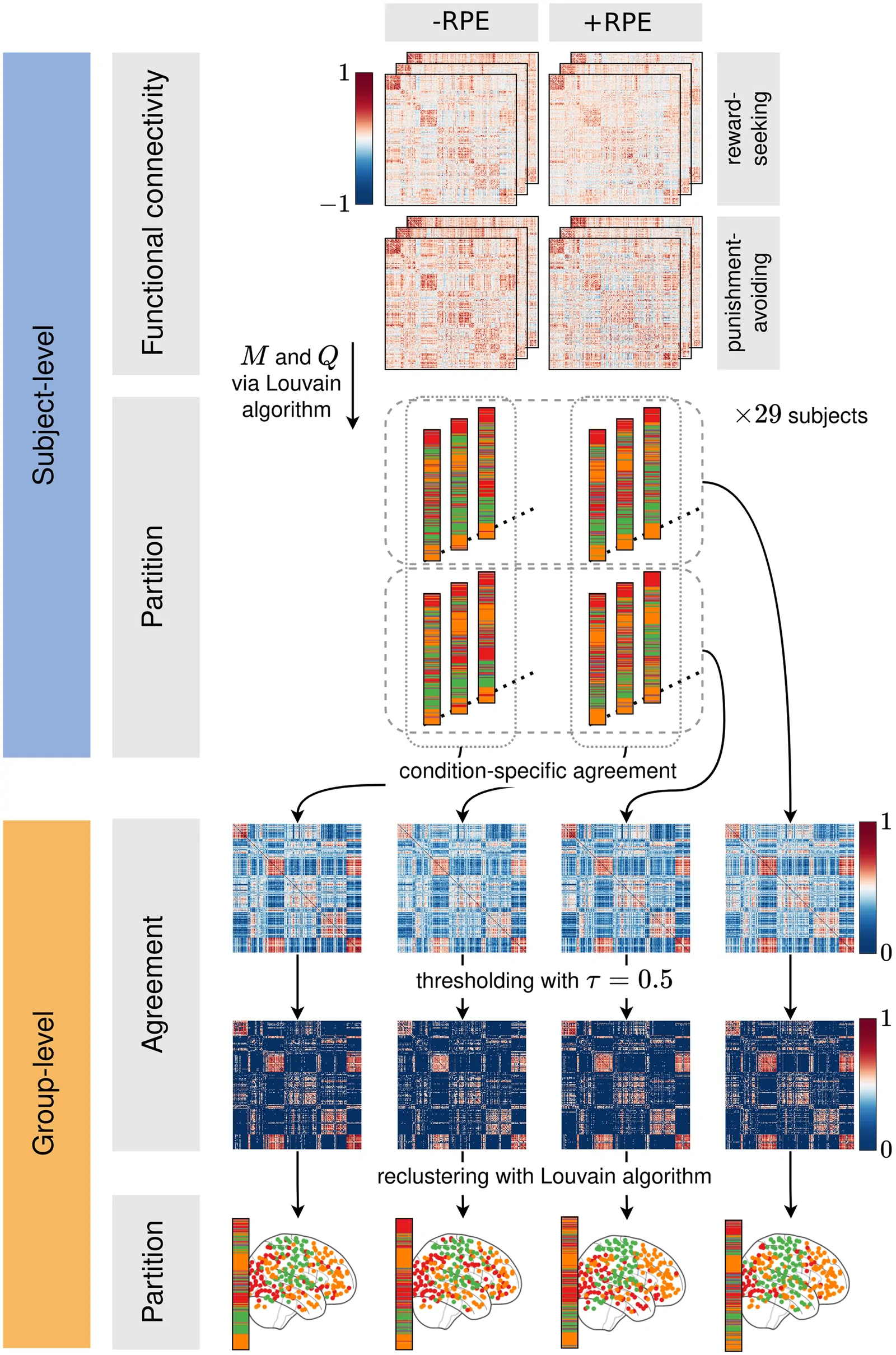
Consensus partitioning pipeline. The multistep procedure employed to calculate the representative network partitions for task contexts and RPE signs. The subject-level connectivity matrices were first clustered via the Louvain algorithm to calculate the network modularity, *Q*, and the community affiliation vectors, *M* (Blondel et al., 2008). Then, the subject-level partitions were grouped into four groups: reward-seeking, punishment-avoiding, positive RPE, and negative RPE. For each group, the agreement matrix was calculated, thresholded, and reclustered, yielding a final context-specific consensus partition.

The representative modular structure for task contexts and RPE signs was calculated in four steps (Figure 7). First, the subject-level community affiliation vectors were aggregated into four groups: reward-seeking, punishment-avoiding, positive RPE, and negative RPE. Each group consisted of two vectors per subject, that is, 58 in total. For example, a reward-seeking group consisted of partitions for both +RPE and −RPE networks in the reward-seeking context. Similarly, a positive RPE group consisted of +RPE networks pooled over the reward-seeking and punishment-avoiding contexts. This pooling procedure allowed us to examine the representative community structure independently for both experimental dimensions. Additionally, the fifth group consisting of partitions from all task contexts was created. It consisted of four vectors per subject, that is, 116 in total. This group was included to represent a modular network structure during RPE processing that is stable across task contexts. Second, an agreement matrix was computed for each group. Third, the agreement matrices were thresholded to remove weak associations; that is, all agreement values below *τ* = 0.5 were set to zero, in accordance with Lancichinetti and Fortunato (2012). Finally, the thresholded agreement matrices were iteratively reclustered with the Louvain algorithm until convergence, and the output affiliation vectors were considered consensus partitions. This procedure yielded four context-specific and one context-independent community vectors. The consensus partitioning procedure was independently repeated for three values of the structural resolution parameter *γ*. Agreement matrices and consensus partitions were calculated using the agreement and consensus_und functions from the bctpy package (version 0.5.2) based on the Brain Connectivity Toolbox (Rubinov & Sporns, 2010).

### Community-Level Agreement

**Figure 8.**
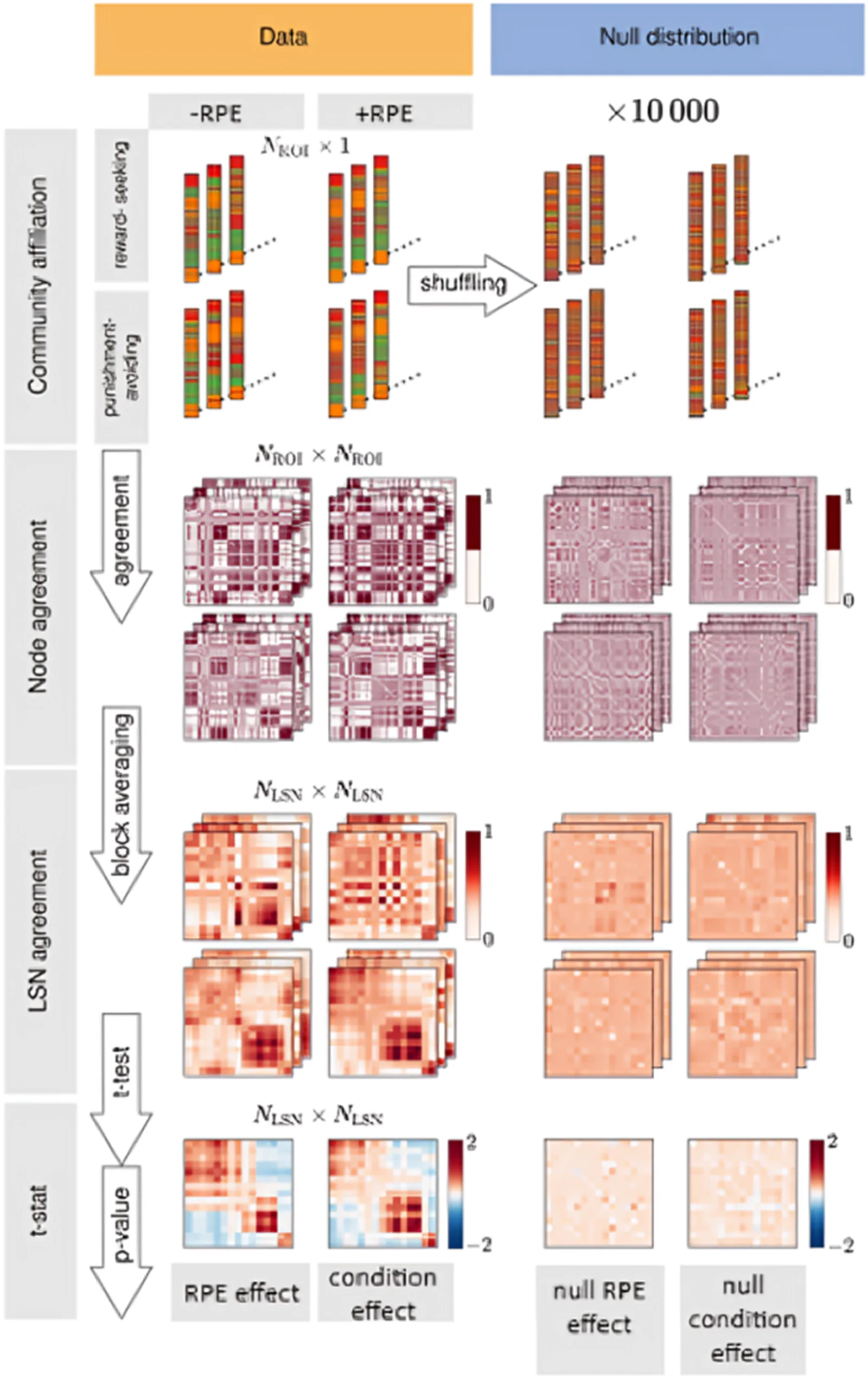
LSN agreement pipeline. The multistep procedure was employed to calculate context-specific changes in the community-level agreement. Subject-level community affiliation vectors were used to calculate node-level agreement matrices, which were block averaged to produce individual LSN agreement matrices. The LSN agreement represents the probability that two randomly selected nodes from reference LSNs belong to the same data-driven community. For each entry in the LSN agreement matrix, two *t* tests were conducted, testing the effect of the task context and the RPE sign. The *t* test significance was tested using the Monte Carlo permutation procedure. The shuffled community affiliation vectors were randomly reordered 10,000 times and subjected to the same analysis procedure as the original vectors to produce a null distribution for both *t* statistics. The *p* values were assigned according to the position of the true *t* statistic within the null distribution.

To investigate context-specific changes in the community-level agreement, individual community affiliation vectors were used to calculate individual node-level agreement matrices (Figure 8). An individual node-level agreement is an *N* ROI × *N* ROI matrix with entries *D*_*ij*_ ∈ {0, 1} indicating whether a pair of nodes belongs to the same data-driven community; this matrix was then condensed into an *N* × *N* LSN matrix by applying block averaging to the node-level agreement values, where “block averaging” refers to averaging these values based on the network division into LSNs (Power et al., 2011). ROIs and LSNs are distinct entities in the network. ROIs represent individual brain regions, while LSNs represent LSNs that emerge from the analysis. In a context-independent consensus partition to create individual LSN agreement matrices:

$$D_{mn}^{\text{LSN}}=\frac{1}{NM}\sum_{i,j=1}^{N_{\text{ROI}}}D_{ij}\delta_{i}^{m}\delta_{j}^{n}$$ (10)

where *N* and *M* are the sizes of communities *n* and *m*, and $\delta_{i}^{m}$ is a community indicator equal to 1 if node *i* belongs to community *m* and 0 otherwise. Then, for each LSN community-level agreement value, *D*_*mn*_, a two-sided paired *t* test was conducted for the task-context and RPE sign effects. The null hypothesis states that the degree of LSN agreement is the same in the reward-seeking and punishment-avoiding contexts for a task-context effect. For the RPE sign effect, the statistical test aims to answer if there is a significant change of LSN agreement when switching between the signaling of positive and negative RPEs.

To determine the significance of the computed *t* statistics, a Monte Carlo permutation procedure was employed (Conrad et al., 2020). Permutation-based procedures represent an alternative approach to significance testing that is especially useful in multidimensional neuroimaging data (Nichols & Holmes, 2002). A total number of 10,000 iterations was performed to obtain a reliable estimate of the null distribution for the computed *t* statistics. For each iteration, the subject-level community allegiance vector, *M*, reflecting the modular network structure, was randomly shuffled to represent a random topology unrelated to the observed functional connections while preserving the number and size of the original communities. These vectors were then subjected to the same procedure employed for the actual dataset consisting of agreement calculation, block-averaging, and statistical testing. Each iteration outputted two null *t*-statistic matrices of size *N LSN* × *N LSN* for both effects of interest. A set of 20,000 of these matrices formed a null distribution of the *t* statistic. *p* values were then calculated based on the null distribution percentile corresponding to the true *t* statistic. An effect was considered significant if the true *t* statistic was lower than the 2.5th or higher than the 97.5th percentile of the null distribution. A two-sided approach was used to account for both increases and decreases in the community-level agreement between task contexts. Permutation-based *p* values were then corrected for multiple comparisons using the FDR method (Benjamini & Hochberg, 1995).

## ACKNOWLEDGMENTS

The study was supported by the National Science Centre, Poland (2020/36/T/HS6/00104). K.B., K.F., and W.D. were supported by *The Excellence Initiative—Research University* awarded to the Nicolaus Copernicus University, and W.D. was partially supported by Polish National Science Center Grant UMO-2016/20/W/NZ4/00354.

## SUPPORTING INFORMATION

Supporting information for this article is available at https://doi.org/10.1162/NETN.a.544.

## AUTHOR CONTRIBUTIONS

Kamil Bonna: Conceptualization; Data curation; Formal analysis; Funding acquisition; Investigation; Methodology; Project administration; Resources; Software; Visualization; Writing – original draft; Writing – review & editing. Oliver James Hulme: Formal analysis; Resources; Supervision; Validation; Writing – review & editing. Simon R. Steinkamp: Writing – review & editing. David Meder: Formal analysis; Methodology; Resources; Supervision; Writing – review & editing. Maria E. C. van der Weij: Writing – review & editing. Włodzisław Duch: Supervision; Writing – review & editing. Karolina Finc: Conceptualization; Formal analysis; Funding acquisition; Investigation; Methodology; Project administration; Resources; Supervision; Validation; Writing – original draft; Writing – review & editing.

## FUNDING INFORMATION

Kamil Bonna, National Science Centre, Award ID: 2020/36/T/HS6/00104.

## DATA AND CODE AVAILABILITY

All code used for neuroimaging and behavioral data processing and statistical data analyses are publicly available at https://github.com/kbonna/decidenet. The raw fMRI data are available from the corresponding author on request.

## Supplementary Material



## TECHNICAL TERMS

Reward prediction error (RPE): The difference between expected and actual outcomes, signaling whether events are better or worse than anticipated.
Large-scale brain networks (LSN): Distributed sets of brain regions that show coordinated activity and support distinct cognitive functions.
Community structure: The partition of a network into groups of nodes that are more strongly connected to each other than to others.
Topological scale (*γ* parameter): A parameter controlling the resolution at which network communities are detected, from coarse to fine.
Beta-series correlation: A method estimating task-related functional connectivity using trial-by-trial activation estimates.
Within- and between-network agreement: Probabilities that brain regions belong to the same community within or across LSNs.
Consensus partition: A representative community structure derived from repeated clustering to identify stable network organization.
Network modularity: A measure of how strongly a brain network is organized into separate, internally cohesive communities.
