# Supplementary material for: Brain network reconfiguration during reward prediction error processing
Source: Netw Neurosci. 2026 Aug 28;10(3):853–83. doi: 10.1162/NETN.a.544 (PMC13569333; doi:10.1162/NETN.a.544)
Supplement: Supplementary file 1 [file netn-10-3-853-s001.pdf]

1. Behavioral modeling **2**
1.1. Bayesian modeling 2
1.2. Markov Chain Monte Carlo 3
1.3. Parameter estimation 4
1.4. Relationship between model parameters and behavioral performance 4
2. fMRI data processing **6**
2.1. Preprocessing 6
2.2. Model-based fMRI 7
2.2.1 First-level GLM 7
2.2.1 Second-level GLM 8
2.2.2 Context-independent RPE processing 9
2.2.3 Context-dependent RPE processing 11
2.3. Analysis of functional brain networks 12
2.3.1 Brain parcellation 12
2.3.2 Beta-series correlation 15
2.3.3 Multi-scale modularity 16
2.3.4 Consensus partition composition for various spatial scales 19

1. Behavioral modeling

1.1. Bayesian modeling

Bayesian modeling provides a principled and flexible way of relating formal cognitive models to behavioral data (Lee & Wagenmakers, 2014). It aims to estimate a set of latent parameters, $\theta$of behavioral models that can be used for inference and model comparison. In Bayesian statistics, parameters are represented as probability distributions instead of single numbers, which preserves the information about their uncertainty. The general principle of Bayesian analysis is to use collected data, X, to update the prior beliefs about parameters to posterior beliefs represented as new probability distributions over the parameters in question. The "updating process" is captured in a Bayes formula:

$$p(\theta \mid X) = \frac{p(X \mid \theta)}{p(X)} p(\theta)$$

where $p(\theta \mid X)$ is a posterior distribution of θ given the data X, $p(X \mid \theta)$ is a probability of observing data X given prior beliefs about θ, $p(\theta)$ is a prior probability distribution, and $p(X)$ is a normalizing constant. Posterior distribution $p(\theta \mid X)$ represents updated belief about parameters and takes into account both prior beliefs and collected data X.

A Bayesian hierarchical latent-mixture (HLM) model was used to perform model selection and parameter estimation. This type of Bayesian model contains parameters representing behavioral parameters like learning rates or precision, and indicator parameters reflecting which competing model is believed to generate the observed data. The hierarchical structure of the model assumes behavioral parameters of

individuals coming from group-level distributions. The latent-mixture structure assumes that observed behavior can arise as a combination of computations from different cognitive processes. The HLM model was estimated using four different models as competing submodels for model comparison: (1) a Prediction Independent Context Independent (PICI) model with a single learning rate independent of the sign of the RPE and outcome valence, (2) a Prediction Independent Context Dependent (PICD) model with two separate learning rates for reward-seeking and punishment-avoiding contexts, (3) a Prediction Dependent Context Independent (PDCI) model with two separate learning rates for positive and negative RPEs, (4) a Prediction Dependent Context Dependent (PDCD) model with four separate learning rates for the RPE signs and task-contexts. Then, a hierarchical model with each submodel as a generative model was estimated for parameter inference.

Weakly informative hyperpriors were set to model the distribution of the learning rates and precision across individuals. Following previous studies, learning rates were assumed to come from a beta distribution (Gershman, 2016). The beta distribution is defined for the continuous interval $\alpha \in [0, 1]$ covering the entire range of possible learning rate values:

$$f(a, b, \alpha) = c_{a,b}\alpha^{a-1}(1-\alpha)^{b-1}$$

where a and b are two non-negative shape parameters. These were set to come from the uniform hyperprior distributions a ~ Uniform(1, 10) and b ~ Uniform(1, 10). Note that both shape parameters were assumed to be greater than 1, since for $a < 1$ and $b < 1$ the beta distribution takes a behaviorally implausible U-shape. The precision parameter β was independently included for each submodel to provide additional flexibility in the model parameter space and avoid potential dependence between competing models. A generative distribution for the precision parameter β was represented as a lognormal distribution. Hyperpriors for lognormal parameter distributions were set to μ ~ Uniform(−2.3, 3.4) for lognormal group mean and σ ~ Uniform(0.01, 1.6) for lognormal group standard deviation. These weakly informative hyperpriors were used in previous studies and derived from the constraints to confine the group mean for the precision parameter within a 0.1 to ~ 30 interval (Meder *et al.*, 2019; Nilsson *et al.*, 2011). The latent-mixture part of the model assumed a subject-specific model indicator variable to represent a latent mixture of four competing submodels. This assumption allowed us to model each subject response pattern independently by using a different mixture of competing submodels. The model indicator variable, z_i, was modeled with a uniformly distributed flat prior representing lack of expectations toward which behavioral model best explains a subjects' response patterns.

1.2. Markov Chain Monte Carlo

Typically, the posterior distribution $p(\theta \mid X)$ can only be calculated analytically for a limited set of simple Bayesian models. For more complex models, the analytical approach becomes ineffective and different solutions are needed. An efficient, computer-driven sampling method known as Markov Chain Monte Carlo (MCMC) has been developed to overcome this issue (Gamerman & Lopes, 2006; Kass *et al.,* 1998). The MCMC method samples the posterior probability distribution $p(\theta \mid X)$ by generating Markov chains. A sufficiently large number of samples in a chain allows for an accurate reproduction of $p(\theta \mid X)$. Here, the MCMC sampling was performed using JAGS software (v4.3.0; http://mcmc-jags.sourceforge.net/) called from MATLAB R2017a via matjags.m script (v1.3.3; https://github.com/msteyvers/matjags). A sampling procedure with 2000 burn-in samples, four chains, and 15,000 samples per chain was used to estimate the probability distribution $p(\theta \mid X)$ of the hierarchical latent-mixture model.

To ensure that the chain samples are unaffected by starting values and come from a stationary distribution, it is common to measure sampling convergence. A popular diagnostic measure – potential scale

reduction, R, was calculated for each model variable, and corresponding chain traceplots were visually inspected to ensure that the procedure converged. $\hat{R}$ combines information on the variation within and between chains to determine whether all chains reflect the same stationary target distribution. Convergence was declared for $\hat{R}$ values less than 1.01. For model selection, a posterior model probability was calculated for each subject using posterior samples of the model indicator variable. PI and PD models were independently compared by marginalizing model indicator variables over subjects and calculating Bayes factors for PICI+PICD (PI model family) models versus PDCI+PDCD (PD model family) models. The Bayes factor is a measure of relative model evidence (marginal likelihood) of two competing hypotheses calculated as:

$$BF_{10} = \frac{p(\theta \mid \mathcal{H}_1)}{p(\theta \mid \mathcal{H}_0)}$$

where subscripts 1 and 0 indicate the two competing hypotheses. Here, these hypotheses refer to PI and PD model families generating observed behavioral data, and θ represents the model indicator variable. An analogous procedure was repeated to compare CI and CD models. A standard interpretation of Bayes factors was used to report levels of evidence (van Doorn *et al*., 2021).

The single best submodel was selected by calculating estimated model frequencies and protected exceedance probabilities (Rigoux *et al.*, 2014) for the model frequency being highest among other submodels. These calculations were performed with the Variational Bayesian Analysis toolbox (v1.9.2; https://github.com/MBB-team/VBA-toolbox), which implements Bayesian model selection for group studies. Model parameters for the winning model were fitted by estimating a reduced hierarchical latent-mixture model. A full model was reduced to the single hierarchical Bayesian model by removing the model indicator variable and all branches representing the alternative competing submodels. Posterior samples for learning rates and precision were used to derive point estimates for these parameters.

1.3. Parameter estimation

The winning PDCI submodel was independently evaluated as a generative model for subjects' behavioral responses. A hierarchical model comprising the branch of the full HLM model without the model indicator variable node was created and sampled using the MCMC sampling technique. Posterior distributions of learning rates and precision nodes enabled estimating individual values of these latent cognitive variables. First, group-level distributions of behavioral parameters were investigated by marginalizing individual distributions over subjects. Learning rates for positive reward prediction errors (RPEs) were higher than learning rates for negative RPEs ($\alpha+ = 0.737 \pm 0.275$; $\alpha- = 0.415 \pm 0.149$). Bayesian hypothesis testing was used to determine whether learning rates for positive RPEs are higher than for negative RPEs (H1) compared to a model in which they are not (H0). Moderate evidence was found in favor of this hypothesis ($BF_{10} = 4.78$). However, after restricting this hypothesis to the 19 subjects with most of their probability mass located over the PDCI model, very strong supporting evidence ($BF_{10} = 37.90$) was found. These results suggest a greater influence of positive than negative RPEs on value estimates, especially in subjects whose behavior is best explained by the PDCI model.

1.4. Relationship between model parameters and behavioral performance

To better understand the behavioral significance of the observed gap between learning rates for positive and negative RPEs, we investigated a possible relationship between the difference in learning rates and the mean number of reversals. A significant negative correlation between the two variables was found ($r = -0.76$; $p <$

0.0001), indicating that the higher difference between the learning rate for positive and negative RPEs was related to the lower reversal tendency. These results suggest that probability matching behavior is reflected by (1) a high learning rate for positive RPEs as subjects are more likely to repeat their choice after the previous correct choice and (2) a relatively low learning rate for negative RPEs as subjects tend to switch their choice after a previous incorrect choice.

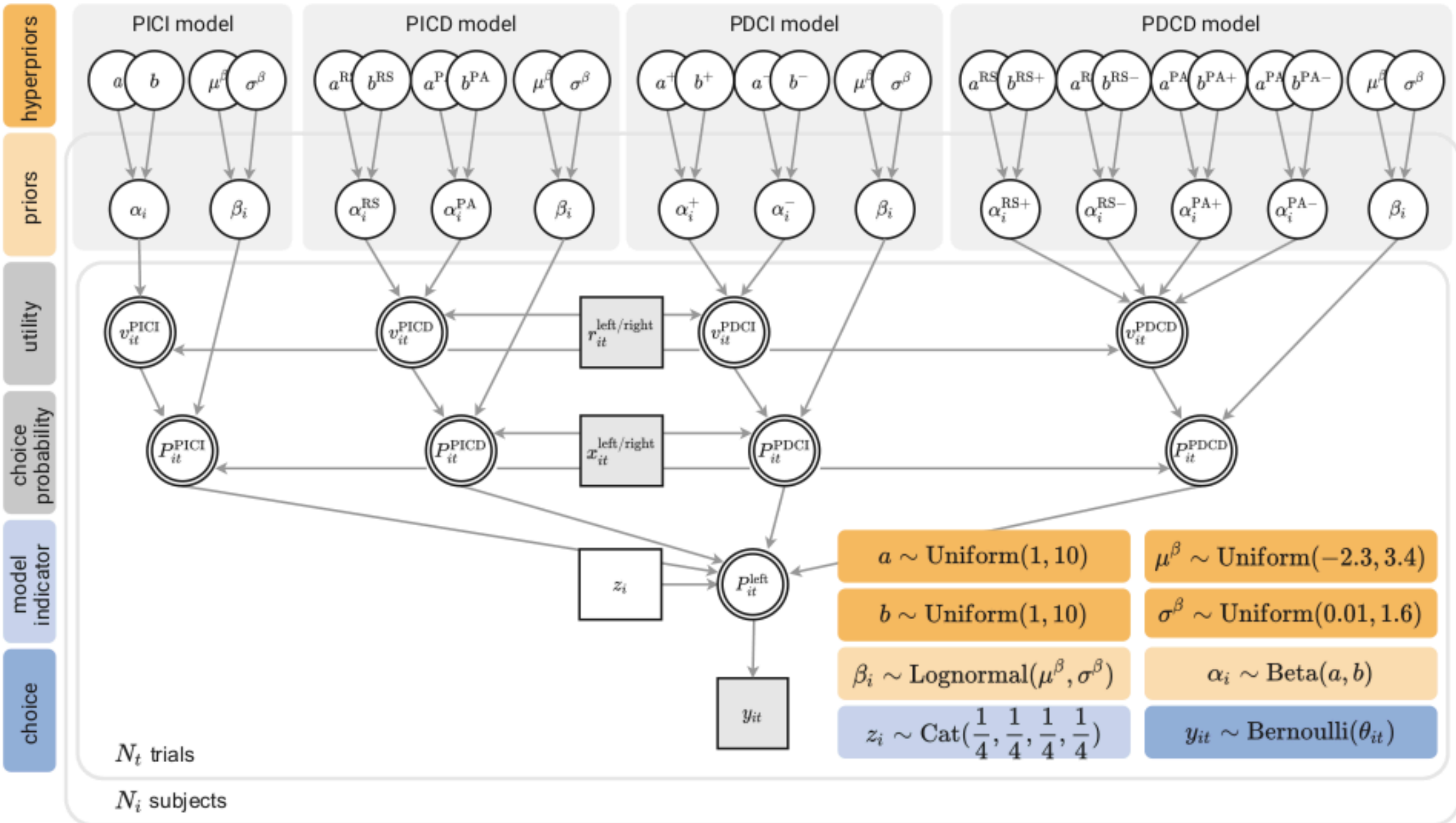

Fig. S1 Hierarchical latent-mixture model. Choice probability for the trial *t* and subject *i* was modeled as a latent mixture of choice probabilities calculated for four different reinforcement learning submodels: PICI with single learning rate for each subject, PICD with two separate learning rates for reward-seeking and punishment-avoiding condition, PDCI with two separate learning rates for positive and negative RPEs and PDCD with four separate learning rates for reward-seeking and punishment-avoiding conditions and positive and negative RPEs. Probabilities for each submodel are approximated by a subject-dependent model indicator variable z_i. Circular nodes represent continuous variables, square nodes represent discrete variables, unshaded nodes represent unobserved variables, shaded nodes represent observed variables, single border nodes represent stochastic variables, and double border nodes represent deterministic variables. Boxes on the left-hand side describe the role of each variable in the model, boxes on the bottom-right present the details of the prior and hyperprior distributions.

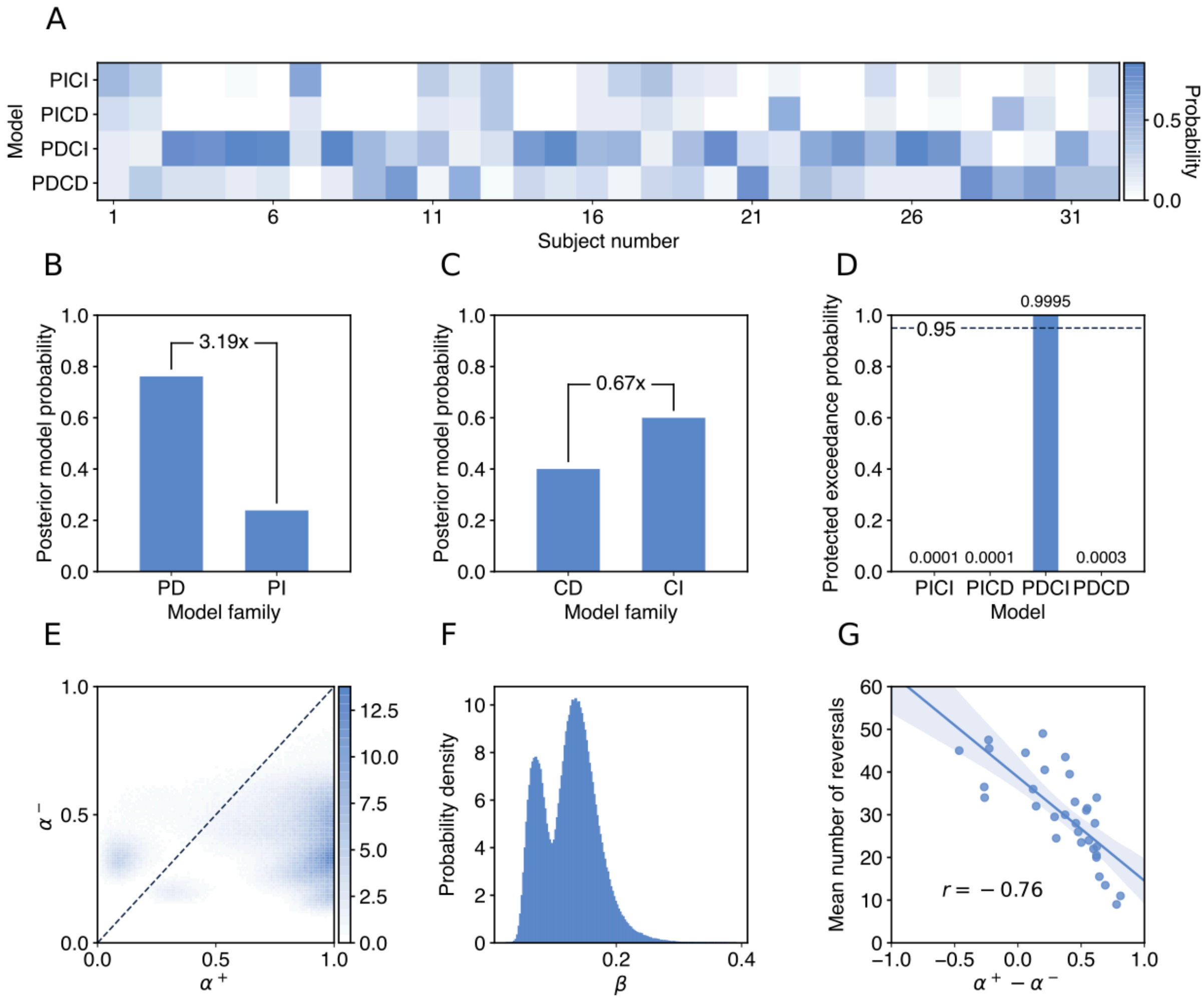

Fig. S2. Model selection and parameter estimation results. (**A**) Posterior distribution of the model indicator variable z_i. Most of the subjects had probability mass over either PDCI (19/32) or PDCD (8/32) models. (**B** and **C**) Posterior distribution of the model indicator variable z_i marginalized over subjects and model classes. Model families: RPE dependent (PD; PDCI and PDCD models); RPE independent (PI; PICI and PICD models); condition dependent (CD; PICD and PDCD models); condition independent (CI; PICI and PDCI models). Numbers over the bars represent Bayes factors for the hypothesis that behavioral responses are better explained by the models with separate learning rates for (**B**) positive and negative RPEs or (**C**) reward-seeking and punishment-avoiding conditions. (**C**) Protected exceedance probabilities for each submodel being more frequent than all other submodels. (**E**) Posterior probability distribution of learning rates for positive, α^+, and negative RPEs, α^-, for the winning PDCI model. The hierarchical model with PDCI submodel as a generative model for subject responses was evaluated independently of the main HLM model. (**F**) Posterior probability distribution of the precision parameter for the winning PDCI model. (**G**) Relationship between the difference in positive and negative learning rates estimated from the PDCI model and a mean number of reversals indicating probability matching behavior.

2. fMRI data processing

2.1. Preprocessing

Neuroimaging data were preprocessed using fMRIprep version 1.4.1 (Esteban *et al*., 2019; Gorgolewski *et al*., 2011). The T1-weighted images were corrected for intensity non-uniformity (INU) with N4BiasFieldCorrection (Tustison *et al*., 2010), distributed with ANTs 2.2.0 (Avants *et al*., 2008), and used further as reference T1-weighed image. The T1w reference was then skull-stripped with a Nipype implementation of the antsBrainExtraction.sh workflow (from ANTs), using OASIS30ANTs as the target template. Brain tissue segmentation of cerebrospinal fluid (CSF), white matter (WM), and gray matter

(GM) was performed on the brain-extracted T1w using FAST (FSL 5.0.9, Zhang *et al*. (2000)). Brain surfaces were reconstructed using recon-all (FreeSurfer 6.0.1, Dale *et al*. (1999)), and the brain mask estimated previously was refined with a custom variation of the method to reconcile ANTs-derived and FreeSurfer-derived segmentation of the cortical gray matter of Mindboggle (Klein *et al.*, 2017). Volume-based spatial normalization to the standard space (MNI152NLin2009cAsym) was performed through nonlinear registration with antsRegistration (ANTs 2.2.0), using brain-extracted versions of both T1w reference and the T1w template. The ICBM 152 Nonlinear Asymmetrical template was selected for spatial normalization (version 2009c; TemplateFlow ID: MNI152NLin2009cAsym; Yoon *et al*. (2009)).

For each of the two fMRI runs per subject, the following preprocessing was performed. First, a reference volume and its skull-stripped version were generated using a custom methodology of fMRIPrep. The blood-oxygen-level dependent reference was then co-registered to the T1w reference using bbregister (FreeSurfer), which implements boundary-based registration (Greve & Fischl, 2009). Co-registration was configured with nine degrees of freedom to account for distortions remaining in the BOLD reference. Head-motion parameters for the BOLD reference were estimated before any spatiotemporal filtering using mcflirt (FSL 5.0.9, (Jenkinson, 2003)). fMRI time series were slice-time corrected using 3dTshift from AFNI 20160207 (Cox & Hyde, 1997).

Then, time series were resampled to surfaces on the fsaverage5 space, their original native space, and standard MNI152NLin2009cAsym space by applying a single composite transform to correct head motion and susceptibility distortions. These resampled time series are referred to as preprocessed time series. First, a reference volume and its skull-stripped version were generated using a custom methodology of fMRIPrep. Several confounding time series were calculated based on the preprocessed time series: framewise displacement (FD), a spatial standard deviation of successive difference images (DVARS), and three region-wise global signals. FD and DVARS were estimated for each functional run, using their implementations in Nipype. The three global signals were extracted from the CSF, WM, and whole-brain masks. Additionally, head-motion estimates calculated during the correction step were stored as confound time series. All head-motion and global signal confounds were expanded with the inclusion of temporal derivatives and quadratic terms (Satterthwaite *et al.*, 2013). Frames that exceeded a threshold of 0.5 mm FD or 1.5 standardized DVARS were marked as motion outliers.

2.2. Model-based fMRI

2.2.1 First-level GLM

The single-subject effect of processing increasing RPEs was modeled with a subject-level general linear model (GLM). Two separate GLMs were created for reward-seeking and punishment-avoiding task conditions. Each GLM was composed of regressors modeling task events and estimated latent decision parameters and nuisance regressors accounting for noise components related to head motion and scanner drift. Task regressors of interest included regressor modeling onset of the outcome phase and its parametric modulation with the estimated trial-wise RPE regressor. Task regressors of no interest modeled other experimental events: onset of the decision and its parametric modulation with the expected probability that chosen option will be correct, left and right button press, trials with a missing response, and offset of the outcome phase (**Fig. S3**). Nuisance regressors included 24 head motion parameters to remove residual movement artifacts and cosine functions derived from the cosine drift model to remove low-frequency artifacts. All task events were modeled as impulse functions with zero duration and convolved with a canonical double-gamma hemodynamic response function (HRF). The first-level contrast was defined as a one-sample

t-test for the RPE regressor effect against the baseline. Resulting statistical parametric maps (SPMs) were used in the second level of the analysis.

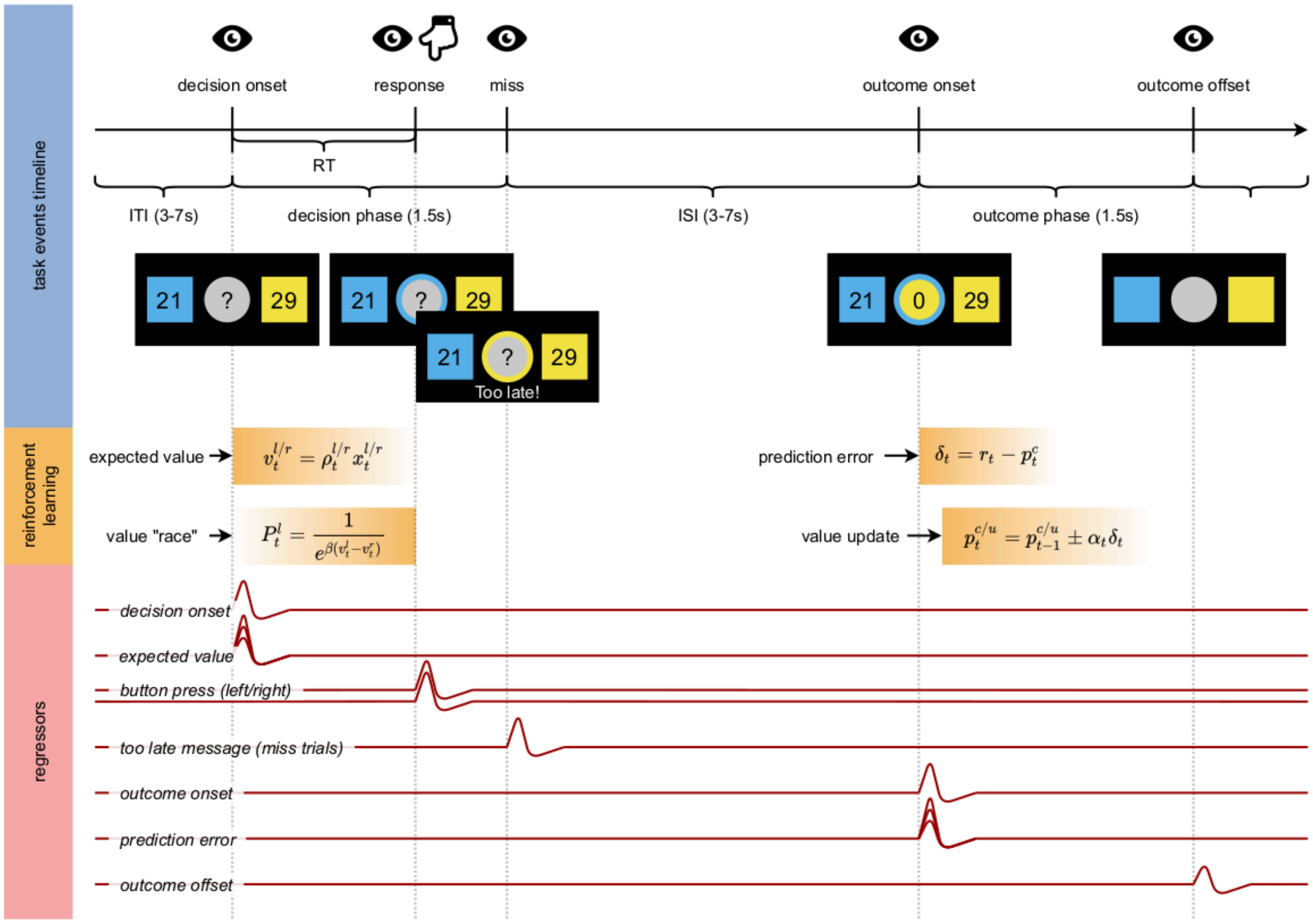

Fig. S3 Event model for a single trial. Task events, latent computational variables, and GLM regressors are shown on a timeline for a single experimental trial. **The top panel** shows a trial timeline with corresponding visual changes (eye icon) and motor responses (hand icon). **The middle pane**l shows latent reinforcement learning variables like expected value and RPEs time-locked to task events. **The bottom panel** presents modeled BOLD responses used in the first-level GLM.

2.2.1 Second-level GLM

The second level of the analysis was performed as a random-effects analysis separately modeling each task condition and within-subject effects. The significance of the two effects of interest on the group level was tested: (1) the combined effect of increasing and decreasing RPEs across both task conditions and (2) the difference in response to increasing and decreasing RPEs between reward-seeking and punishment-avoiding conditions. A threshold of $p < 0.0001$ with false discovery rate (FDR) correction was used for the former effect, accounting for its high statistical power compared with the latter effect. This stringent thresholding was imposed to reduce the number of significant clusters and improve the readability of the statistical maps. A threshold of $p < 0.001$ with FDR correction was used for the latter effect. An additional threshold for a cluster size of 20 connected voxels was used in either case.

Differences in response to RPEs between reward-seeking and punishment-avoiding conditions cannot be unambiguously interpreted since statistical testing of this effect relies on testing the difference between slopes of the regression between voxel activity and the RPE. For example, a significant response for increasing RPEs in reward-seeking compared to the punishment-avoiding condition may be related to one

of the three effects: (1) for both conditions, activity is correlated with increasing RPE, but for the reward-seeking condition the relationship is significantly stronger, (2) for both conditions activity is correlated with decreasing RPE, but for the reward-seeking condition, the relationship is significantly weaker or (3) activity is correlated with increasing RPE in the reward-seeking condition and correlated with decreasing RPE in the punishment-avoiding condition. A post hoc test on the cluster level was performed to discriminate between these effects. Response profiles were extracted for each subject, task condition, and significant cluster exhibiting increased or decreased response to RPEs in the reward-seeking condition compared to the punishment-avoiding condition. These profiles were calculated as first-level parameter estimates, i.e., GLM beta values, for peak voxel for the RPE regressor effect. Then, average beta values across subjects were calculated for both conditions. To further confirm the significant change in response profiles, a paired t-test between reward-seeking and punishment-avoiding betas was performed for all clusters.

2.2.2 Context-independent RPE processing

We wanted to identify brain regions in which activity increases with increasing RPEs and decreases with decreasing RPEs. We used individually calculated RPEs to construct RPE regressors, modeling latent RPE computation time-locked to the outcome onset. Our first aim was to identify brain regions encoding increasing and decreasing RPEs regardless of the task condition. These regions form the core of the learning network enabling the processing of positive and negative feedback information used to update the values of chosen stimuli. A broad network of regions in which activity was correlated with increasing and decreasing RPEs was found (**Fig. S4**). For increasing RPEs, significant activity was found in clusters in the vmPFC, superior temporal gyrus, bilateral orbitofrontal cortex, bilateral putamen, left postcentral gyrus, right amygdala, and VS, left posterior cingulate cortex (PCC), left angular gyrus, right precentral gyrus, and left middle temporal gyrus (**Table S1**; increasing RPE). Other significant clusters were located in the left caudal middle frontal gyrus, bilateral planum temporale, left parahippocampal gyrus, and right superior frontal gyrus. For decreasing RPEs, significant activity was found in clusters in the left dorsomedial cingulate cortex, bilateral anterior insula (aINS), right pars opercularis, left dorsolateral prefrontal cortex (dlPFC), left cerebellum, left precentral gyrus, and left superior frontal gyrus (**Table S1**; decreasing RPE). Generally, activity related to increasing RPEs was more widespread than activity related to decreasing RPEs.

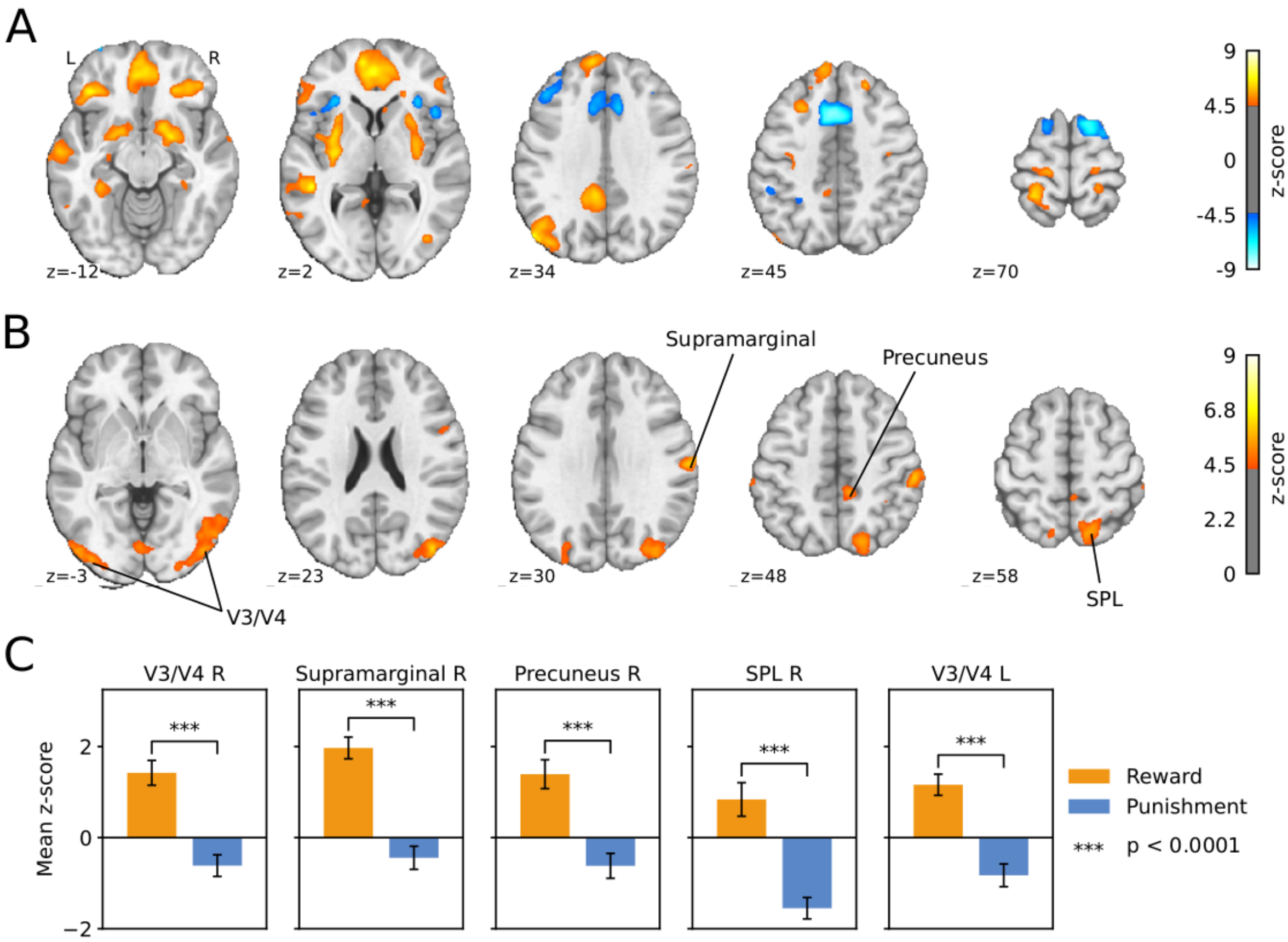

Fig. S4 Model-based fMRI results. (**A**) Brain regions processing increasing and decreasing RPEs. Orange regions exhibit increased activity with increasing RPE in both task conditions. Blue regions decrease their BOLD response with increasing RPE in both task conditions. Statistical maps were FDR corrected with a threshold $p < 0.0001$. (**B**) Brain regions responding differently to RPEs in rewards-seeking and punishment-avoiding conditions. For orange clusters slope between voxel activity and RPE was higher in reward-seeking compared to punishment-avoiding conditions. (**C**) Post-hoc test for the clusters in B (only five clusters with the highest z-scores are shown). First-level parameter estimates (z-scores) for the RPE regressor were extracted individually for each cluster, subject, and condition. Each region in B increased its activity with increasing RPE in the reward-seeking condition and decreased its activity with increasing RPE in the punishment-avoiding condition (paired t-test; $p<0.0001$). Abbreviations: SPL - superior temporal lobule.

2.2.3 Context-dependent RPE processing

We wanted to test whether RPE signaling in essential parts of an increasing RPE network like striatum and vmPFC undergo valence-related changes. To investigate differences in RPE processing between task conditions, we evaluated a second-level GLM testing for higher response to increasing RPEs in reward-seeking than punishment-avoiding conditions. A set of brain regions was found for which the regression slope between voxel activity and RPE was higher in the reward-seeking compared to punishment-avoiding condition (**Fig. S4B**). These regions included bilateral visual areas V3 and V4, right supramarginal gyrus, right superior parietal lobule, right precuneus, right primary visual cortex, and right precentral gyrus (Table 4.2). None of these regions were part of the core RPE processing network. Since differences in response to the RPE between reward-seeking and punishment-avoiding conditions cannot be unequivocally interpreted, a *post hoc* test was performed for all significant clusters by extracting individual subject and condition response profiles as first-level parameter estimates for the peak voxel. Positive activity scaling with increasing RPEs in reward-seeking condition and negative activity scaling with increasing RPEs in punishment-avoidance condition was found in all seven clusters (paired t-test; p<0.0001; **Fig. S4C**). There were no brain regions with significantly higher responses to increasing RPEs in the punishment-avoiding condition.

Region	L/R	X	Y	Z	Z-score peak	Cluster Size (mm^3)
			Increasing PE			
Ventromedial Prefrontal Cortex	L	-3	48	-1	7.23	30397
Superior Temporal Gyrus	L	-48	-39	3	6.93	1417
Lateral Orbitofrontal Cortex	L	-39	33	-12	6.79	5071
Putamen	L	-30	-12	6	6.63	8473
Postcentral Gyrus	L	-27	-42	66	6.62	1732
Amygdala, Ventral Striatum, Putamen	R	21	0	-12	6.52	8347
Posterior Cingulate Cortex	L	-6	-45	38	6.47	9985
Angular Gyrus	L	-54	-75	34	6.32	9009
Precentral Gyrus	R	24	-24	59	6.30	2803
Middle Temporal Gyrus	L	-60	-12	-15	6.18	2425
Caudal Middle Frontal Gyrus	L	-27	24	48	6.07	1039
Lateral Orbitofrontal Cortex	R	27	36	-12	6.00	3213
Planum Temporale, Supramarginal Gyrus	R	45	-36	17	5.86	3244
Parahippocampal Gyrus	L	-33	-39	-12	5.80	1417
Precentral Gyrus	L	-15	-27	73	5.69	1039
Superior Frontal Gyrus	R	21	39	55	5.69	1386
Planum Temporale	L	-57	-33	17	5.69	1669
Middle Temporal Gyrus	L	-57	-57	-1	5.39	850
Precentral Gyrus	L	-6	-18	52	5.39	787
Postcentral Gyrus	L	-39	-24	52	5.28	913
			Decreasing PE			
Dorsomedial Cingulate Cortex, SMA	L	-6	12	48	8.18	14647
Anterior Insula	L	-33	21	10	6.71	1543
Anterior Insula	R	30	24	6	6.43	756
Pars Opercularis	R	45	18	3	5.93	787
Dorsolateral Prefrontal Cortex	L	-45	30	34	5.89	2803
Cerebellum	L	-33	-60	-26	5.81	630
Precentral Gyrus	L	-30	-3	59	5.62	913
Superior Frontal Gyrus	L	-18	6	69	5.55	882

Table S1 Between-condition differences in RPE signaling. Regions exhibiting differences in RPE processing between reward-seeking and punishment-avoiding conditions.

Region	L/R	X	Y	Z	Z-score peak	Cluster Size (mm3)
Increasing PE Reward-seeking > Punishment-avoiding						
V3 / V4	R	42	-81	24	6.28	13608
Supramarginal Gyrus	R	57	-27	52	6.22	3843
V3 / V4	L	-48	-81	-1	5.64	3402
Superior Parietal Lobule	R	21	-72	45	5.60	4410
Precuneus	R	12	-39	52	5.56	1071
V1	R	3	-78	3	5.42	1921
Precentral Gyrus	R	54	9	17	5.29	693
Increasing PE Punishment-avoiding > Reward-seeking						
no significant clusters						

Table S2 Between-condition differences in RPE signaling. Regions exhibiting differences in RPE processing between reward-seeking and punishment-avoiding conditions.

2.3. Analysis of functional brain networks

2.3.1 Brain parcellation

One of the first steps in brain network analysis is dividing the brain into regions that will be considered nodes of the functional network (Sporns, 2013). Each specific division is called brain parcellation or brain atlas. One frequently used brain parcellation in resting-state and task-based fMRI studies has been introduced by Power *et al*. (2011). It consists of 264 regions of interest defined based on a meta-analysis of activation patterns from multiple studies. Each ROI is modeled as a 5mm sphere around the specific MNI coordinates. Power's parcellation additionally includes the division of the 264 ROIs into 13 large-scale networks (LSNs): auditory, cerebellar, somatomotor, cingulo-opercular, default mode, memory, ventral attention, dorsal attention, fronto-parietal, salience, subcortical, uncertain and visual. This division allows examining the dynamic interplay between LSNs in various experimental conditions. However, Power's parcellation does not contain separate RPE-related networks; therefore, it cannot be directly used to study how the reward system interacts with the rest of the brain during reinforcement learning. To overcome this issue, Power's ROIs were combined with regions from the recent meta-analysis on neural representations of RPE valence (Fouragnan *et al*., 2018). This procedure allows studying effects specific for networks signaling RPEs while still preserving reference to well-known LSNs. Moreover, a recent meta-analysis provided evidence that reward-related brain regions form a stable network were observed even during rest (Huckins *et al.*, 2019).

The following procedure was employed to create extended brain parcellations. First, two RPE-related networks were created based on cluster centers for the valence analysis in a recent meta-analysis on neural correlates of RPEs (Table 2 in Fouragnan *et al.* (2018)). The network signaling positive RPEs was created from regions exhibiting higher BOLD activation for positive than negative RPEs (pattern A (ii) in Fouragnan *et al*. (2018)). Conversely, the network signaling negative RPEs consisted of regions with a higher BOLD response for negative than positive RPEs (pattern A(i) in Fouragnan *et al.* (2018)). Second, for each

cluster center, a new ROI was created using one of the three strategies based on its distance to the closest ROI in Power's atlas (d):

- If d > 10mm, there was no overlap between the 5mm sphere created at the cluster center and existing ROIs, so a new ROI was created and added to the ROIs set without any modification of the existing base parcellation.
- If 10mm > d > 5mm, there was a slight overlap between the 5mm sphere created at the cluster center and the closest Power's ROI (no more than 31% of sphere volume overlapped). In this case, the closest Power's ROI was shifted into the location of the activation likelihood estimation (ALE) cluster center and renamed according to the assignment to one of the RPE-related networks.
- If d < 5mm there was a significant overlap between the 5mm sphere created at the cluster center and the closest Power's ROI (more than 31% of sphere volume overlapped). In this case, the original Power's ROI was retained at its original location and relabelled according to the assignment to one of the RPE-related networks.

All newly created ROIs were initially modeled as 5mm spheres around specific MNI coordinates. Radii of four striatal ROIs – left and right pallidum and left and right ventral striatum were decreased to 4mm to avoid overlap between spheres (**Table S3**). The extended brain parcellation consisted of 272 ROIs divided into 15 networks (13 LSNs from Power *et al*. (2011) and 2 RPE networks). Four ROIs from the uncertainty network – MNI coordinates (-31, -10, -36), (-56, -45, -24), (8, 41, -24), and (52, -34, -27) – were further excluded due to signal dropout in some participants. The final parcellation consisted of N ROI = 268 regions of interest.

Region	R/L	X	Y	Z	r(mm)	Original network	Strategy
ROIs signaling positive prediction errors (+PE)							
Ventrolateral orbitofrontal cortex	R	32	44	-10	5	Uncertain	shifted
Ventral striatum	L	-12	8	-4	4	-	created
Posterior cingulate cortex	L	0	-36	26	5	Memory	shifted
Medial prefrontal cortex	L	-2	46	20	5	Default mode	shifted
Ventral striatum	R	8	8	-2	4	-	created
Dorsomedial prefrontal cortex	L	-6	-56	14	5	Default mode	shifted
Ventromedial prefrontal cortex	L	-2	42	0	5	Default mode	shifted
ROIs signaling negative prediction errors (−PE)							
Anterior insula	L	-32	22	-4	5	Salience	shifted
Middle frontal gyrus	R	32	14	56	5	Fronto-parietal	relabelled
Inferior parietal lobule	R	40	-48	42	5	Fronto-parietal	shifted
Superior temporal sulcus	R	54	-43	22	5	Ventral attention	relabelled
Fusiform area	L	-42	-60	-9	5	Dorsal attention	relabelled
Pallidum	R	12	8	4	4	Subcortical	shifted
Dorsolateral prefrontal cortex	R	40	34	30	5	Salience	shifted
Dorsolateral prefrontal cortex	L	-42	25	30	5	Fronto-parietal	relabelled
Middle temporal gyrus	R	60	-28	-6	5	Default mode	shifted
Inferior parietal lobule	L	-38	-48	42	5	Dorsal attention	shifted
Thalamus	L	-6	-26	8	5	Subcortical	shifted
Anterior insula	R	32	24	-2	5	Salience	shifted
Pallidum	L	-14	6	2	4	Subcortical	shifted
Dorsomedial prefrontal cortex	R	20	50	4	5	-	created
Dorsomedial orbitofrontal cortex	R	38	58	-2	5	-	created
Precentral cortex	L	-52	0	34	5	-	created
Habenula	R	2	-20	-18	5	-	created
Amygdala	R	18	-6	-12	5	-	created
Middle frontal gyrus	R	38	4	32	5	-	created
Middle frontal gyrus	L	-28	12	60	5	Fronto-parietal	shifted
Dorsomedial cingulate cortex	R	5	23	37	5	Salience	relabelled

Table S3 Between-condition differences in RPE signaling. RPE signaling ROIs created from ALE clusters from Fouragnan et al. (2018).

2.3.2 Beta-series correlation

Task-related functional connectivity was estimated using a beta-series correlation method introduced by Rissman *et al*. (2004). The method's name comes from the GLM parameter beta, representing the linear coefficient reflecting the contribution of the modeled signal into an observed BOLD response. The BSC method quantifies event-to-event fluctuations in the activity of different brain areas. These fluctuations allow for estimating statistical dependency between regional activations for different event types.

In BSC, each trial is modeled separately in GLM. Then, a series of beta maps representing brain activations for a series of events was used to calculate condition-specific connectivity evoked by the task. An alternative methodology to calculate task-evoked functional connectivity is psychophysiological interaction analysis (PPI, Friston *et al*., 1997). However, it has been suggested that the BSC method can have more statistical power than the PPI method when applied to event-related designs with many trials and short event durations (Cisler *et al*., 2014). The "single-trial-versus-other-trial" approach introduced by Mumford *et al*. (2012) was used to compute activation beta maps. This approach assumes creating a separate GLM for each trial in which the trial of interest is modeled as a separate regressor, and all other trials are modeled as a single nuisance regressor. Simulations have shown that the "single-trial-versus-other-trial" approach produces more accurate estimates of trial-wise activation patterns (Mumford *et al*., 2012).

For each trial, a separate GLM was created. The GLM consisted of:

- One regressor modeling a trial of interest as an event of duration 0s (typically used to model very short cognitive processes) time-locked to the onset of the outcome phase, convolved with a standard SPM HRF.
- Two regressors modeling the rest of the trials of interest as events of duration 0s time-locked to the onset of the outcome phase convolved with an HRF. One regressor modeled trials with positive RPEs (gain in reward-seeking condition or no-loss in punishment-avoiding condition). The other modeled trials with negative RPEs (no-gain in the reward-seeking condition or loss in the punishment-avoiding condition).
- Six regressors modeling the events of no interest: decision onset, decision onset modulated with expected probability for side for being correct, decision onset for missed trials, left button press, right button press, and decision offset for missed trials.
- Twenty-four head motion parameter regressors modeling motion-related noise
- Cosine functions modeling low-frequency scanner drift.

The GLM was high-pass filtered with a cut-off frequency of 128 Hz and fitted to participants' data using the `FirstLevelModel` class from the Nistats package. The output of this procedure consisted of a three-dimensional beta map for each trial, indicating the voxel-wise estimate of neuronal activity evoked during this trial (**Fig. S.5**). After GLM estimation, beta values for each trial were averaged in each of 268 ROIs producing 268 beta-series per subject and task condition. Beta-series were then z-transformed and separated by the sign of the RPE of each trial. This procedure gave four beta-series for each participant: +RPE trials in the reward-seeking condition, −RPE trials in the reward-seeking condition, +RPE trials in the punishment-avoiding condition, and −RPE trials in the punishment-avoiding condition. Beta-series were then correlated using Pearson's correlation coefficient, generating an $N\ ROI \times N\ ROI$ symmetrical correlation matrix for each subject, task condition, and trial-wise RPE sign. Correlation values were then converted to z-scores using Fisher's z-transform. Transformed matrices represented the final subject-level estimate of the functional connectivity pattern evoked by the specific task condition and type of trial.

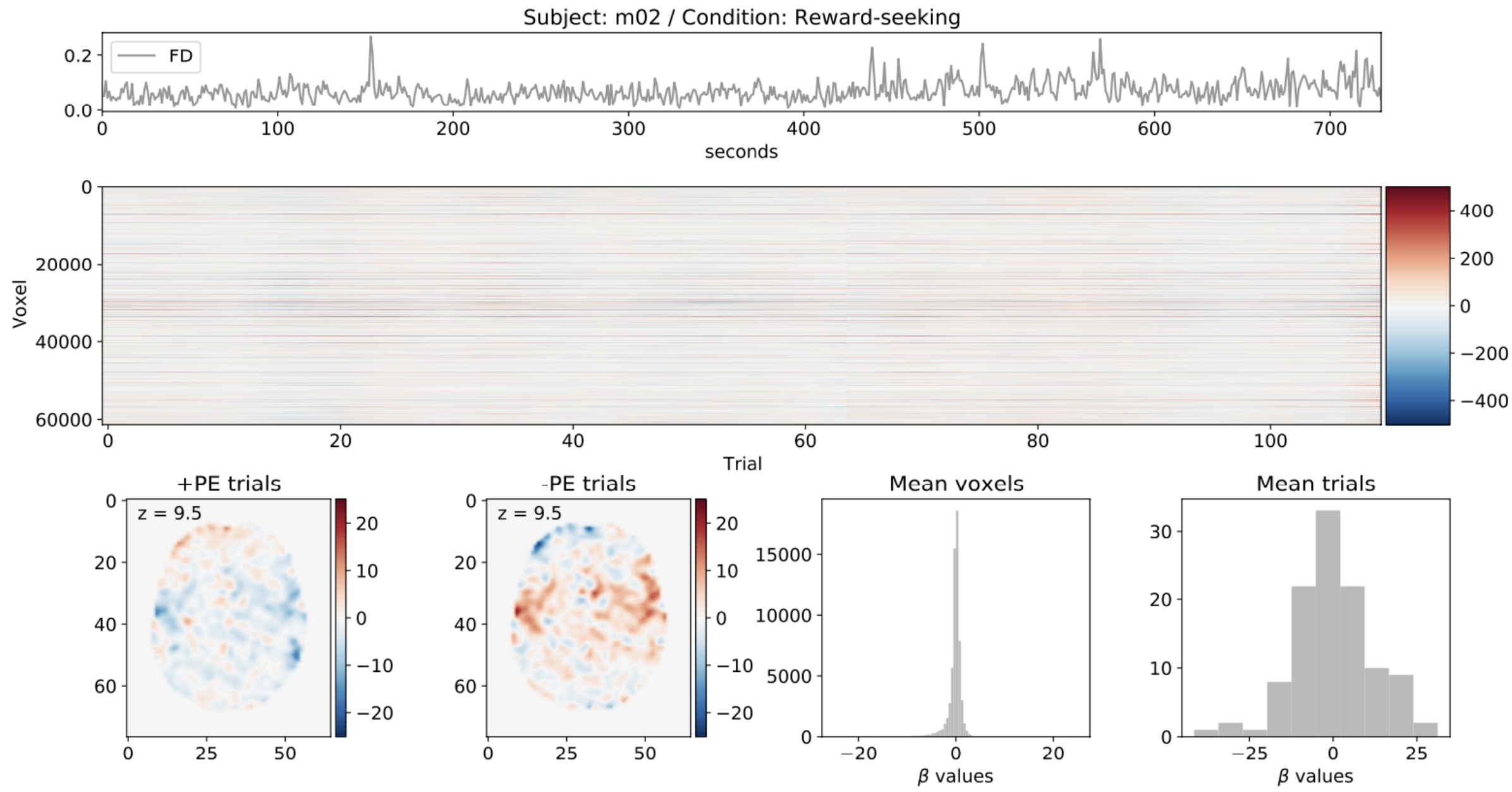

Fig. S5 Example beta maps summary for single subject and task condition. Different characteristics of trial-wise beta maps in the BSC analysis. The top panel shows the time course of the FD. The middle panel shows the carpet plot of beta estimates – the x-axis corresponds to 110 trials of the probabilistic reversal learning (PRL) task, the y-axis corresponds to all voxels within the brain mask, color indicates trial-evoked activation. Two bottom-left panels show a coronal slice for z=9.5 for mean brain activation during +RPE and -RPE trials. Two bottom-right panels show beta-value distributions averaged across trials and voxels.

2.3.3 Multi-scale modularity

Multi-scale modularity analysis was performed by introducing a structural resolution parameter, γ, into the modularity function:

$$Q(\gamma) = \sum_{ij}(A_{ij} - \gamma P_{ij})\delta(\sigma_i \sigma_j)\,,$$

where A_{ij} is the connection strength between nodes *i* and *j*, P_{ij} is the expected connection strength according to the appropriate null model, σ_i indexes the community to which node *i* is assigned, and δ is the Kronecker delta function. Tuning the structural resolution parameter allows uncovering communities of different sizes otherwise mathematically undetectable using the standard modularity function (Fortunato & Barthelemy, 2007; Reichardt & Bornholdt, 2006). Specifically, lower values of γ result in fewer larger communities, and higher values produce more and smaller communities. At the lower end of the γ range, all network nodes are placed within one community, whereas at the higher end, there are as many communities as there are nodes. Different heuristics have been proposed to find an appropriate sampling of structural resolution parameter space. For example, similarity measures between partitions have been used to minimize the variability in the results of modularity maximization techniques (Doron *et al.*, 2012; Lancichinetti *et al.*, 2009). This choice has been guided by the intention to describe network reorganization with reference to well-known LSNs. Two constraints have been imposed on the analyzed topological scales:

1. The number of detected modules and mean module size should be similar to the reference partition. In other words, large γ values generating partitions with many singletons, i.e., single node communities should be excluded from the analysis.
2. The detected communities should exhibit high similarity to the reference partition while preserving a high degree of inter-individual variability. High similarity to the reference partition justifies the interpretation of the results in terms of interactions between a priori LSNs. High inter-individual variability ensures that the analysis will be more sensitive to topological changes between task conditions and RPE signs.

To calculate community structure properties across topological scales, the structural resolution parameter space was sampled by varying γ from 0.05 to 3 with a step of 0.05. For each value of γ, the Louvain modularity maximization algorithm was used to quantify the optimal network structure (Blondel *et al*., 2008). Negative connections were separately included in the modularity function and treated asymmetrically as suggested in Rubinov and Sporns (2011):

$$Q^{asym}(\gamma) = Q^{+}(\gamma) + \frac{v^{-}}{v^{-} + v^{+}} Q^{-}(\gamma) \, ,$$

where Q ± (γ) are modularity values calculated separately for positive and negative connections, and v ± = ij A ± ij are total connection strengths, i.e., costs for positive and negative connections. For each functional network, the Louvain algorithm was run 100 times, and the partition with the highest value of *Q asym* (γ) was kept for further analysis. Several metrics were computed to inspect different topological scales (**Fig. S6**): (1) a mean number of communities, singletons, and non-singletons (communities with more than one node), (2) mean community size, (3) mean similarity between data-driven partitions, and (4) mean similarity between reference partition and data-driven partitions. Partition similarity was computed as the normalized mutual information between community partition vectors (Meilă, 2007).

The mean number of data-driven communities was closest to 14 reference communities for γ = 1.5 (average 14.59 communities per partition). For γ > 1.5, there was a steep increase in the number of singletons, whereas the number of non-singletons rose to 21.85 for γ = 2 and plateaued with a further increase in structural resolution. The average data-driven community size was above 100 nodes per partition for 0 < γ < 0.9 and rapidly declined for γ ~ 1. For γ = 1.65, the average community size was closest to the average reference community size (18.86 nodes per community for data-driven partitions; 19.14 nodes per community for reference partition). The similarity between partitions measured as normalized mutual information increased as a function of γ. For 0.15 ≤ γ ≤ 2.1, data-driven partitions exhibited higher similarity to themselves than to reference partition. The ratio between data-driven partition similarity, MI_{d-d} , and similarity between reference and data-driven partition, MI_{d-d} = 1.85). $M\,I_{d-r}$, was highest for γ = 0.4.

According to the first heuristic, the analyzed γ range should include structural resolutions for which the number of communities and the average size of the community resembles characteristics of the reference partition. This consideration suggests including γ ~ 1.5 since the size and number of data-driven communities were closest to the size and number of reference communities for this topological scale. On the other hand, the second heuristic suggests including γ ~ 0.5 because partitions observed for this topological scale exhibit the optimal similarity to the reference partition while preserving a high degree of inter-individual variability. For γ > 2 the number of singletons starts to dominate non-singleton communities, and relative inter-individual variability decreases compared to similarity to the reference partition, suggesting that this part of the structural resolution scale should be excluded from further analysis. Considering both heuristics, the final range 0.5 ≤ γ ≤ 1.5 was adopted for all subsequent analyses. Whereas

dense sampling of this range (e.g., with a step of 0.05) would allow tracing how observed effects smoothly appear and disappear when changing a topological resolution, it would also be computationally expensive and challenging to describe statistically. Therefore, a sparse sampling of γ range was employed, enabling to focus on three distinct topological scales: γ = 0.5 representing the largest scale of few "super-communities" consisting of multiple LSNs, γ = 1 representing the most frequently studied intermediate scale, and γ = 1.5 representing a finer scale approximately resembling well-known divisions into LSNs.

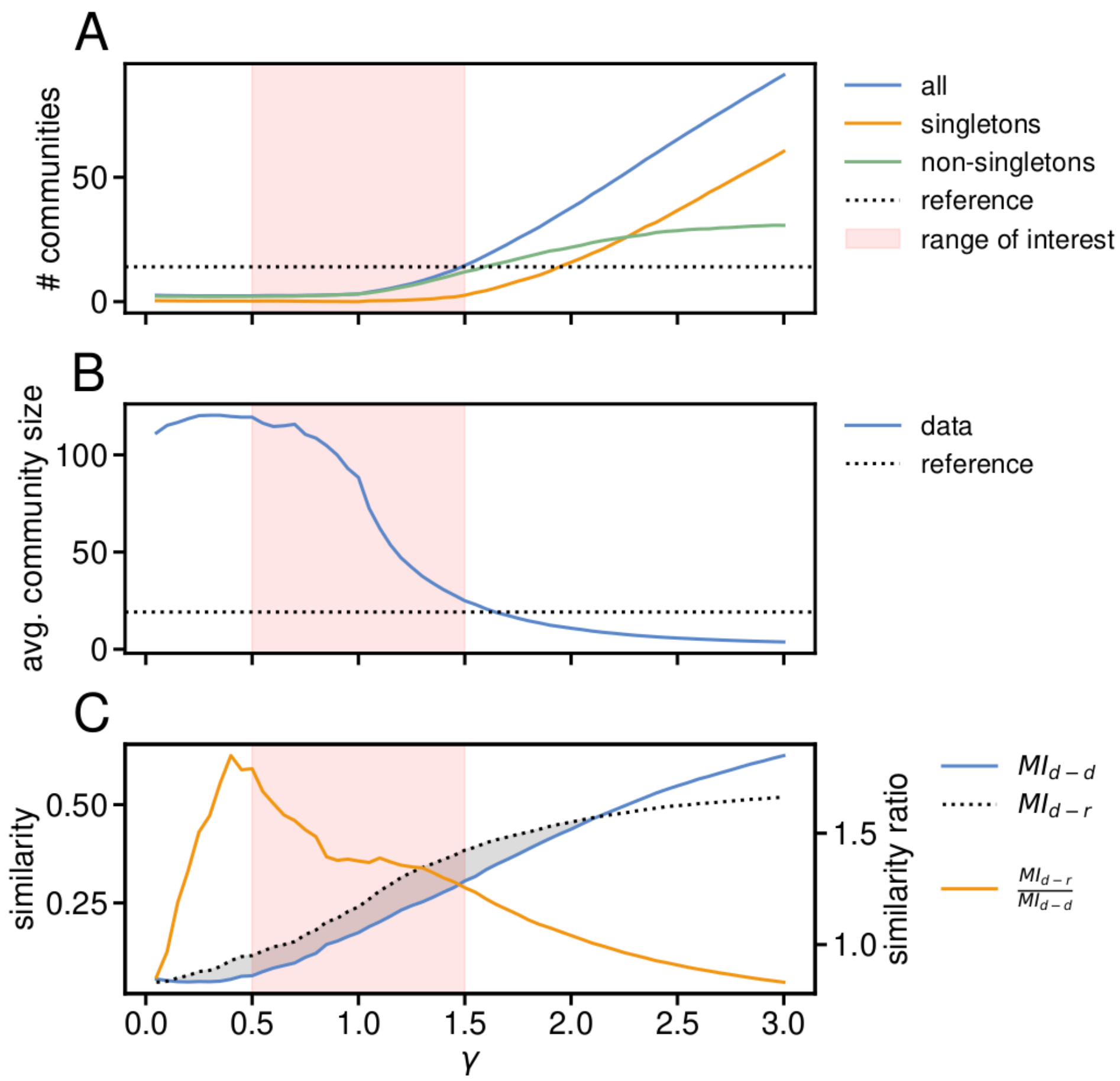

Fig. S6 Structural resolution parameter analysis. The structural resolution parameter, γ, was varied from 0.05 to 3 with a step of 0.05. For each value of γ, optimal community structures for empirical networks were calculated using 100 repetitions of the Louvain algorithm (Blondel et al., 2008). (**A**) The number of all communities, singleton communities, and non-singleton communities. The reference partition based on Power ROIs described in section 4.6.1 has 14 communities. (**B**) Mean data-driven community size compared with the average size of 19.14 ROIs per community for reference partition. (**C**) Average similarity between data-driven partitions and between reference and data-driven partitions. The gray area indicates γ range for which partitions are more similar to the reference than to themselves. The orange line reflects the optimal trade-off between high similarity to reference partition and high inter-individual variability in data-driven partitions. MI_{d-d} - average mutual information between all pairs of data-driven partitions; MI_{d-r} - average mutual information between data-driven partitions and reference partition.

2.3.4 Consensus partition composition for various spatial scales

Module	Condition	n	Large-scale networks														
			+PE	DM	−PE	FP	MEM	SAL	UNC	SUB	VAT	CER	SOM	CO	AUD	DAT	VIS
Task-Visual	+PE	144	0,71	0,70	0,86	1,00	0,75	0,71	0,83	0,40	0,25	0,25	0,00	0,00	0,00	0,33	0,65
	−PE	137	0,86	0,76	0,76	0,95	0,75	0,64	0,78	0,20	0,50	0,25	0,00	0,00	0,00	0,33	0,45
	RS	128	1,00	0,83	0,71	0,95	0,75	0,71	0,74	0,50	0,50	0,00	0,00	0,00	0,00	0,00	0,06
	PA	136	0,71	0,59	0,86	1,00	0,75	0,64	0,70	0,40	0,13	0,25	0,00	0,00	0,00	0,33	0,74
	all	141	0,86	0,70	0,86	1,00	0,75	0,71	0,83	0,30	0,25	0,25	0,00	0,00	0,00	0,33	0,55
Sensory	+PE	118	0,14	0,24	0,14	0,00	0,25	0,29	0,13	0,60	0,63	0,75	1,00	1,00	1,00	0,67	0,35
	−PE	131	0,14	0,24	0,24	0,05	0,25	0,36	0,22	0,80	0,50	0,75	1,00	1,00	1,00	0,67	0,55
	RS	140	0,00	0,17	0,29	0,05	0,25	0,29	0,26	0,50	0,50	1,00	1,00	1,00	1,00	1,00	0,94
	PA	132	0,29	0,41	0,14	0,00	0,25	0,36	0,30	0,60	0,88	0,75	1,00	1,00	1,00	0,67	0,26
	all	127	0,14	0,30	0,14	0,00	0,25	0,29	0,17	0,70	0,75	0,75	1,00	1,00	1,00	0,67	0,45
vmPFC	+PE	6	0,14	0,06	0,00	0,00	0,00	0,00	0,04	0,00	0,13	0,00	0,00	0,00	0,00	0,00	0,00

Table S4 Consensus partition composition for structural resolution parameter γ = 0.5. Cell values correspond to a percentage of reference network regions belonging to the consensus module. Cell color intensity reflects the underlying percentage value. The reference partition consists of 13 well-known LSNs from the Power atlas (Power et al., 2011) and two RPE-signaling networks (see section 4.6.1). The third column shows the consensus module size. Task condition abbreviations: +RPE - positive reward prediction error trials, –RPE - negative reward prediction error trials, RS - risk-seeking condition, PA - punishment-avoiding condition. Reference LSNs abbreviations: DM - default mode, FP - fronto-parietal, MEM - memory, SAL - salience, UNC - uncertain, SUB - subcortical, VAT - ventral attention, CER - cerebellar, SOM - somatomotor, CO - cingulo-opercular, AUD - auditory, DAT - dorsal attention, VIS - visual.

Module	Condition	n	Large-scale networks														
			+PE	DM	−PE	FP	MEM	SAL	UNC	SUB	VAT	CER	SOM	CO	AUD	DAT	VIS
Task-Visual	+PE	96	0,57	0,06	0,71	0,86	0,75	0,64	0,52	0,10	0,00	0,00	0,03	0,00	0,00	0,44	0,84
	−PE	98	0,57	0,06	0,76	0,90	0,75	0,71	0,57	0,10	0,00	0,25	0,00	0,00	0,00	0,33	0,81
	RS	87	0,29	0,02	0,67	0,86	0,75	0,57	0,48	0,10	0,00	0,00	0,03	0,00	0,00	0,33	0,81
	PA	107	0,57	0,07	0,81	1,00	0,75	0,71	0,57	0,10	0,00	0,25	0,03	0,00	0,00	0,33	0,94
	all	94	0,57	0,04	0,71	0,86	0,75	0,71	0,48	0,10	0,00	0,00	0,03	0,00	0,00	0,33	0,84
Default Mode	+PE	75	0,43	0,85	0,19	0,14	0,00	0,14	0,43	0,30	0,50	0,00	0,00	0,00	0,00	0,00	0,00
	−PE	70	0,43	0,85	0,10	0,10	0,00	0,00	0,43	0,30	0,50	0,00	0,00	0,00	0,00	0,00	0,00
	RS	76	0,71	0,87	0,19	0,14	0,00	0,14	0,43	0,10	0,50	0,00	0,00	0,00	0,00	0,00	0,00
	PA	72	0,43	0,91	0,14	0,00	0,00	0,07	0,39	0,30	0,50	0,00	0,00	0,00	0,00	0,00	0,00
	all	74	0,43	0,87	0,14	0,14	0,00	0,07	0,43	0,30	0,50	0,00	0,00	0,00	0,00	0,00	0,00
Sensory	+PE	97	0,00	0,09	0,10	0,00	0,25	0,21	0,04	0,60	0,50	1,00	0,97	1,00	1,00	0,56	0,16
	−PE	100	0,00	0,09	0,14	0,00	0,25	0,29	0,00	0,60	0,50	0,75	1,00	1,00	1,00	0,67	0,19
	RS	105	0,00	0,11	0,14	0,00	0,25	0,29	0,09	0,80	0,50	1,00	0,97	1,00	1,00	0,67	0,19
	PA	89	0,00	0,02	0,05	0,00	0,25	0,21	0,04	0,60	0,50	0,75	0,97	1,00	1,00	0,67	0,06
	all	100	0,00	0,09	0,14	0,00	0,25	0,21	0,09	0,60	0,50	1,00	0,97	1,00	1,00	0,67	0,16

Table S5 Consensus partition composition for structural resolution parameter γ = 1. Consensus partition composition for structural resolution parameter γ = 1. For abbreviations and additional description see Table S4.

References

Avants, B. B., Epstein, C. L., Grossman, M., and Gee, J. C. (2008). Symmetric diffeomorphic image registration with cross-correlation: evaluating automated labeling of elderly and neurodegenerative brain. *Medical Image Analysis*, 12(1):26–41.

Blondel, V. D., Guillaume, J.-L., Lambiotte, R., and Lefebvre, E. (2008). Fast unfolding of communities in large networks. *Journal of Statistical Mechanics: Theory and Experiment,* 2008(10):P10008.

Cisler, J. M., Bush, K., and Steele, J. S. (2014). A comparison of statistical methods for detecting context-modulated functional connectivity in fmri. *Neuroimage*, 84:1042– 1052.

Cox, R. W. and Hyde, J. S. (1997). Software tools for analysis and visualization of fmri data. *NMR in Biomedicine: An International Journal Devoted to the Development and Application of Magnetic Resonance In Vivo*, 10(4-5):171–178.

Dale, A. M., Fischl, B., and Sereno, M. I. (1999). Cortical surface-based analysis: I. segmentation and surface reconstruction. *Neuroimage*, 9(2):179–194.

Doron, K. W., Bassett, D. S., and Gazzaniga, M. S. (2012). Dynamic network structure of interhemispheric coordination. *Proceedings of the National Academy of Sciences*, 109(46):18661–18668.

Esteban, O., Markiewicz, C. J., Blair, R. W., Moodie, C. A., Isik, A. I., Erramuzpe, A., Kent, J. D., Goncalves, M., DuPre, E., Snyder, M., et al. (2019). fMRIPrep: a robust preprocessing pipeline for functional mri. *Nature Methods*, 16(1):111–116.

Fouragnan, E., Retzler, C., and Philiastides, M. G. (2018). Separate neural representations of prediction error valence and surprise: Evidence from an fMRI meta-analysis. *Human Brain Mapping*, 39(7):2887–2906.

Fortunato, S. and Barthelemy, M. (2007). Resolution limit in community detection. *Proceedings of the National Academy of Sciences*, 104(1):36–41.

Friston, K., Buechel, C., Fink, G., Morris, J., Rolls, E., and Dolan, R. J. (1997). Psychophysiological and modulatory interactions in neuroimaging. *Neuroimage,* 6(3):218–229.

Gamerman, D. and Lopes, H. F. (2006). Markov chain Monte Carlo: stochastic simulation for Bayesian inference. *CRC Press*.

Gorgolewski, K., Burns, C. D., Madison, C., Clark, D., Halchenko, Y. O., Waskom, M. L., and Ghosh, S. S. (2011). Nipype: a flexible, lightweight and extensible neuroimaging data processing framework in python. *Frontiers in Neuroinformatics*, 5:13.

Greve, D. N. and Fischl, B. (2009). Accurate and robust brain image alignment using boundary-based registration. Neuroimage, 48(1):63–72.

Huckins, J. F., Adeyemo, B., Miezin, F. M., Power, J. D., Gordon, E. M., Laumann, T. O., Heatherton, T. F., Petersen, S. E., and Kelley, W. M. (2019). Reward- related regions form a preferentially coupled system at rest. *Human Brain Mapping*, 40(2):361–376.

Jenkinson, M. (2003). Fast, automated, n-dimensional phase-unwrapping algorithm. *Magnetic Resonance in Medicine: An Official Journal of the International Society for Magnetic Resonance in Medicine*, 49(1):193–197.

Kass, R. E., Carlin, B. P., Gelman, A., and Neal, R. M. (1998). Markov chain monte carlo in practice: a roundtable discussion. *The American Statistician*, 52(2):93–100.

Klein, A., Ghosh, S. S., Bao, F. S., Giard, J., Häme, Y., Stavsky, E., Lee, N., Rossa, B., Reuter, M., Chaibub Neto, E., et al. (2017). Mindboggling morphometry of human brains. *PLoS Computational Biology*, 13(2):e1005350.

Lancichinetti, A., Fortunato, S., and Kertész, J. (2009). Detecting the overlapping and hierarchical community structure in complex networks. *New Journal of Physics*, 11(3):033015.

Meder, D., Rabe, F., Morville, T., Madsen, K. H., Koudahl, M. T., Dolan, R. J., Siebner, H. R., and Hulme, O. J. (2019). Ergodicity-breaking reveals time optimal decision making in humans. arXiv preprint arXiv:1906.04652.

Mumford, J. A., Turner, B. O., Ashby, F. G., and Poldrack, R. A. (2012). Deconvolving bold activation in event-related designs for multivoxel pattern classification analyses. *Neuroimage*, 59(3):2636–2643.

Nilsson, H., Rieskamp, J., and Wagenmakers, E.-J. (2011). Hierarchical bayesian parameter estimation for cumulative prospect theory. *Journal of Mathematical Psychology*, 55(1):84–93.

Lee, M. D. and Wagenmakers, E.-J. (2014). Bayesian cognitive modeling: A practical course. Cambridge university press.

Power, J. D., Cohen, A. L., Nelson, S. M., Wig, G. S., Barnes, K. A., Church, J. A., Vogel, A. C., Laumann, T. O., Miezin, F. M., Schlaggar, B. L., et al. (2011). Functional network organization of the human brain. *Neuron*, 72(4):665–678.

Reichardt, J. and Bornholdt, S. (2006). Statistical mechanics of community detection. *Physical Review E*, 74(1):016110.

Rigoux, L., Stephan, K. E., Friston, K. J., & Daunizeau, J. (2014). Bayesian model selection for group studies—revisited. *Neuroimage*, 84, 971-985.

Rissman, J., Gazzaley, A., and D'Esposito, M. (2004). Measuring functional connectivity during distinct stages of a cognitive task. *Neuroimage*, 23(2):752–763.

Rubinov, M. and Sporns, O. (2011). Weight-conserving characterization of complex functional brain networks. *Neuroimage*, 56(4):2068–2079.

Satterthwaite, T. D., Elliott, M. A., Gerraty, R. T., Ruparel, K., Loughead, J., Calkins, M. E., Eickhoff, S. B., Hakonarson, H., Gur, R. C., Gur, R. E., et al. (2013). An improved framework for confound regression and filtering for control of motion artifact in the preprocessing of resting-state functional connectivity data. *Neuroimage*, 64:240–256.

Sporns, O. (2014). Contributions and challenges for network models in cognitive neuroscience. *Nature Neuroscience*, 17(5):652–660.

Tustison, N. J., Avants, B. B., Cook, P. A., Zheng, Y., Egan, A., Yushkevich, P. A., and Gee, J. C. (2010). N4itk: improved n3 bias correction. *IEEE Transactions on Medical Imaging*, 29(6):1310–1320.

van Doorn, J., van den Bergh, D., Böhm, U., Dablander, F., Derks, K., Draws, T., Etz, A., Evans, N. J., Gronau, Q. F., Haaf, J. M., et al. (2021). The jasp guidelines for conducting and reporting a bayesian analysis. *Psychonomic Bulletin & Review*, 28(3):813–826.

Yoon, U., Fonov, V. S., Perusse, D., Evans, A. C., Group, B. D. C., et al. (2009). The effect of template choice on morphometric analysis of pediatric brain data. *Neuroimage*, 45(3):769–777.

Zhang, Y., Brady, J. M., and Smith, S. (2000). Hidden markov random field model for segmentation of brain MR image. In Medical Imaging 2000: Image Processing, volume 3979, pages 1126–1137. International Society for Optics and Photonics.
